# Activated L-Arginine/Nitric Oxide Pathway in Pediatric Cystic Fibrosis and Its Association with Pancreatic Insufficiency, Liver Involvement and Nourishment: An Overview and New Results

**DOI:** 10.3390/jcm9062012

**Published:** 2020-06-26

**Authors:** Folke Brinkmann, Beatrice Hanusch, Manfred Ballmann, Sebene Mayorandan, Alexander Bollenbach, Kristine Chobanyan-Jürgens, Kathrin Jansen, Anjona Schmidt-Choudhury, Nico Derichs, Dimitrios Tsikas, Thomas Lücke

**Affiliations:** 1 University Children’s Hospital, Ruhr University, 44791 Bochum, Germany; folke.brinkmann@rub.de (F.B.); manfred.ballmann@med.uni-rostock.de (M.B.); kathrin.jansen@rub.de (K.J.); a.schmidt-choudhury@klinikum-bochum.de (A.S.-C.); luecke.thomas@rub.de (T.L.); 2 Paediatric Clinic, University Medicine Rostock, 18057 Rostock, Germany; 3 Department of Paediatrics, Hannover Medical School, 30623 Hannover, Germany; Sebene.Mayorandan@ukmuenster.de (S.M.); Kristine.Chobanyan-Juergens@med.uni-heidelberg.de (K.C.-J.); arzt@kinderpneumologie-derichs.de (N.D.); 4 Department of Paediatrics, University Clinic Münster, 48149 Münster, Germany; 5 Institute of Toxicology, Core Unit Proteomics, Hannover Medical School, 30623 Hannover, Germany; bollenbach.alexander@mh-hannover.de (A.B.); tsikas.dimitros@mh-hannover.de (D.T.); 6 Department of Clinical Pharmacology and Pharmacoepidemiology, Heidelberg University Hospital, 69120 Heidelberg, Germany; 7 Department of General Pediatrics, Neuropediatrics, Metabolism, Gastroenterology, Nephrology, Center for Pediatric and Adolescent Medicine, University Hospital Heidelberg, 69120 Heidelberg, Germany; 8 Pediatric Clinical-Pharmacological Trial Center (paedKliPS), Center for Pediatric and Adolescent Medicine, University Hospital Heidelberg, 69120 Heidelberg, Germany; 9 KinderPneumologieDerichs, Pediatric Pneumology and Allergology, CFTR & Pulmonary Research Center, 30173 Hannover, Germany

**Keywords:** cystic fibrosis, nitric oxide, L-arginine, nitric oxide synthases, pancreatic disease, nutritional status, malnutrition, liver disease

## Abstract

Cystic fibrosis (CF; OMIM 219700) is a rare genetic disorder caused by a chloride channel defect, resulting in lung disease, pancreas insufficiency and liver impairment. Altered L-arginine (Arg)/nitric oxide (NO) metabolism has been observed in CF patients’ lungs and in connection with malnutrition. The aim of the present study was to investigate markers of the Arg/NO pathway in the plasma and urine of CF patients and to identify possible risk factors, especially associated with malnutrition. We measured the major NO metabolites nitrite and nitrate, Arg, a semi-essential amino acid and NO precursor, the NO synthesis inhibitor asymmetric dimethylarginine (ADMA) and its major urinary metabolite dimethylamine (DMA) in plasma and urine samples of 70 pediatric CF patients and 78 age-matched healthy controls. Biomarkers were determined by gas chromatography–mass spectrometry and high-performance liquid chromatography. We observed higher plasma Arg (90.3 vs. 75.6 µM, *p* < 0.0001), ADMA (0.62 vs. 0.57 µM, *p* = 0.03), Arg/ADMA ratio (148 vs. 135, *p* = 0.01), nitrite (2.07 vs. 1.95 µM, *p* = 0.03) and nitrate (43.3 vs. 33.1 µM, *p* < 0.001) concentrations, as well as higher urinary DMA (57.9 vs. 40.7 µM/mM creatinine, *p* < 0.001) and nitrate (159 vs. 115 µM/mM creatinine, *p* = 0.001) excretion rates in the CF patients compared to healthy controls. CF patients with pancreatic sufficiency showed plasma concentrations of the biomarkers comparable to those of healthy controls. Malnourished CF patients had lower Arg/ADMA ratios (*p* = 0.02), indicating a higher NO synthesis capacity in sufficiently nourished CF patients. We conclude that NO production, protein-arginine dimethylation, and ADMA metabolism is increased in pediatric CF patients. Pancreas and liver function influence Arg/NO metabolism. Good nutritional status is associated with higher NO synthesis capacity and lower protein-arginine dimethylation.

## 1. Introduction

Cystic fibrosis (CF; OMIM 219700) is an autosomal recessively inherited disease affecting about 1:3300 to 1:4800 newborns in Germany [1]. It is caused by mutations in the cystic fibrosis transmembrane conductance regulator (CFTR), an epithelial chloride channel, leading to a thickening of secretions in both the airway epithelium and the pancreatic duct. The severity of lung disease is often life-limiting in CF. Exocrine and endocrine pancreatic insufficiency and secondary liver involvement are further crucial factors contributing to disease severity [2,3]. More than 80% of CF patients are already born with exocrine pancreas insufficiency due to obstruction of small pancreatic ducts, where CFTR is needed to secrete fluids and bicarbonate from pancreas into duodenum. CF patients produce thickened pancreatic juice, which is no longer able to buffer acidic pH levels of gastric acid [4,5]. Malabsorption is a result of exocrine pancreas insufficiency, which is commonly treated with the administration of pancreatic enzymes, the addition of fat-soluble vitamins and hypercaloric nutrition [4,6]. Chronic inflammation of the pancreas is often seen in CF patients, as well as structural changes that often lead to CF-related diabetes later in life [7]. Liver disease in CF patients is also caused by the thickening of bile. About 5% of CF patients require liver transplantation in the course of their life [8].

Energy requirements of CF patients exceed those of healthy individuals due to the increased work of breathing and systemic inflammation. Therefore, hypercaloric nutrition is recommended in all CF patients [9]. Nutritional status is positively associated with the survival of CF patients and it is recommended for them to remain above the 50th percentile of weight-for-length or body mass index (BMI). Nutrition and growth should be monitored closely in CF care, as a decrease in fat-free body mass is associated with a progression of lung disease in children and adults [9].

The nutritionally semi-essential amino acid L-arginine (Arg) can be produced in adequate amounts endogenously by healthy humans beyond infancy and is involved in numerous pathways, including protein synthesis and neuronal functioning [10,11]. Arg is the physiological precursor of nitric oxide (NO), a pleiotropic endogenous signaling molecule involved in vasodilatation, in the innate immune system [12], in neurotransmission, platelet function, and the differentiation and proliferation of stem cells [13,14]. Nitrite and nitrate are major metabolites of NO [15]. Asymmetric dimethylarginine (ADMA) is an endogenous Arg metabolite and inhibitor of NO synthase (NOS) catalyzed synthesis from Arg [16].

ADMA is eliminated by the kidney partially non-metabolized and partially after metabolization predominantly in kidney, liver, lung, pancreas and brain. The most important enzyme responsible for the metabolism of ADMA is dimethylarginine dimethylaminohydrolase (DDAH). This enzyme hydrolyzes ADMA to dimethylamine (DMA) and L-citrulline [17,18]. It is estimated that about 80% of daily endogenously produced ADMA is metabolized by DDAH and is excreted in the urine as DMA, with the remaining 20% being excreted in the urine as ADMA [19]. Urinary DMA is therefore a useful measure of whole-body ADMA synthesis [19].

Based on the concentrations of members of the Arg/NO pathway measured in health and disease, imbalances in the Arg/NO pathway have been connected to certain diseases in the cardiovascular and neuronal systems [20]. Alterations in the Arg/NO pathway have been observed in pediatric patients with inborn metabolic diseases, as well as in pediatric patients with diabetes type I and pediatric attention deficit hyperactivity disorder (ADHD) patients [21,22,23,24]. Yet, members of the Arg/NO pathway, notably ADMA in plasma and urine, were found to decrease from infancy to adulthood [25]. High circulating and low urinary ADMA concentrations are cardiovascular risk factors in adults, but not yet in children, indicating considerable differences in the Arg/NO pathway between adults and children.

Alterations in the Arg metabolism have been reported to be associated with reduced NO synthesis in CF patients [26,27,28,29]. The plasma Arg/ADMA ratio is considered a valuable measure of NO synthesis capacity [26,30,31]. Lower Arg and higher ADMA concentrations in the blood, i.e., a low NO synthesis capacity, may contribute to increased airway obstruction due to elevated smooth muscle tone, presumably resulting from locally low NO synthesis and/or bioavailability. ADMA has been found in higher concentration in the sputum of CF patients compared to controls and might contribute to insufficient NO synthesis by inhibiting NOS activity [26]. Similarly, elevated ADMA concentrations have been found in the breath condensate of children with CF compared to healthy controls [27]. Reduced fractionally exhaled NO (FENO) rates are found during pulmonary exacerbation, with antibiotic treatment increasing FENO [26,28]. ADMA levels in sputum behave differently; they are high during exacerbation and lower in restitution [26,28]. Additionally, higher arginase activity, which hydrolysis Arg to L-ornithine and urea and competes with NOS activity for their shared substrate Arg, was measured in the sputum of CF patients, with the arginase activity being even further elevated during exacerbations [32]. Independent of the lung function, the nutritional status in CF patients has been described to be associated with the level of NO synthesis. Thus, Engelen et al. found higher NO production in CF patients with nutritional failure [29]. Furthermore, in anorexia nervosa patients, elevated FENO levels have been described [33], while in another study on anorexia nervosa patients diminished Arg concentrations were measured in plasma [34].

As data on Arg/NO metabolism in CF with regard to pancreatic and liver health are scarce and nutritional failure might as well influence Arg/NO metabolism, these parameters were analyzed in CF patients. The aim of the present study was to determine the status of Arg/NO pathway in plasma and urine of pediatric CF patients and to test for potential associations with pancreas sufficiency, liver involvement and nourishment. This study might help evolve new early markers with regard to diagnosing and treating these common problems in CF care.

## 2. Experimental Section

### 2.1. Subjects

For the prospective clinical trial, 70 patients (42.9% male; Table 1) with CF, between five and 17 years of age (median, 11.8 years; Table 1), were recruited at the Hannover Medical School either during yearly follow-up or during hospitalization for intravenous antibiotic therapy. Diagnosis of CF was either verified with repeated pathological sweat-tests and/or genetical analysis. CF patients under the age of five or infected by MRSA, Burkholderia cepacia or extended-spectrum β-lactamases were excluded from the study. We included 78 healthy controls (50% male; Table 1) aged between five and 17 years (median, 11.3 years; Table 1), who underwent small elective surgery at the Hannover Medical School or elective control gastroscopies of asymptomatic patients at the University Children’s Hospital in Bochum. Written informed consent was given by parents or legal guardians. The study was approved by the Ethics Committees of the Hannover Medical School and Ruhr-University Bochum.

Clinical parameters of CF patients were measured during routine examination and obtained from health record. Body mass index percentiles (BMIp) were calculated and classified as described by Kromeyer-Hauschild et al. [35]. Nutritional failure was classified as BMIp < 10. Since BMIp does not correlate with the height of children, percentiles of body height were calculated additionally [36]. Liver involvement was classified by sonographic examination of liver and spleen with signs of modification in liver morphology and splenomegaly or hepatomegaly in combination with laboratory parameters: cholinesterase (CHE) < 4 kU/L; prothrombin time < 70% or albumin < 35 g/L. Systolic blood pressure (SBP) and diastolic blood pressure (DBP) were measured by the Riva-Rocci method.

### 2.2. Biochemical Analyses

Venous blood was drawn from non-fasted children in ethylenediaminetetraacetic acid (EDTA) Monovettes (Sarstedt, Nümbrecht, Germany), put on ice, centrifuged immediately, and the plasma samples were stored at −80 °C until analysis. Spot urine samples were collected during routine check-up and stored at −80 °C until further analysis. FENO was measured during yearly check-up for CF patients via NIOX^®^ (Circassia Pharmaceuticals, UK former Aerocrine, Sweden) and reported as parts per billion (ppb). Age-corrected FENO was further classified as low, normal or high using reference FENO data [37]. Low FENO was defined as < 3rd percentile, and high FENO was defined as > 95th.

All biochemical parameters were analyzed by using previously reported validated methods and by considering analyte-specific features, including blood and urine collection, centrifugation, sample storage and sample work-up. Study samples were analyzed alongside quality control samples for all biochemical parameters, as described elsewhere for the individual analytes (see below). All biochemical parameters were determined with the required accuracy (bias ≤ ± 20%) and precision (relative standard deviation, ≤20%).

Citrulline was determined using a commercially available amino acid analyzer model Biochrom 30Plus (Laborservice Onken GmbH, Gründau, Germany) based on ion-exchange high-performance liquid chromatography (HPLC) and post-column derivatization with ninhydrin [22,24]. As NO production in vivo is not reliably measurable, nitrite and nitrate were used as surrogate measures of NO synthesis [38,39]. Nitrate and nitrite in plasma and urine samples were determined simultaneously by gas chromatography–mass spectrometry (GC–MS) as described elsewhere [40,41,42]. Arg in plasma as well as ADMA in plasma and urine were analyzed with GC–MS/MS as described previously [43]. DMA and creatinine were measured in urine by GC–MS as described by us earlier [44,45,46]. The urinary excretion of the NO metabolites nitrite and nitrate, of ADMA and its metabolite DMA was corrected for creatinine excretion and these data are reported as µM analyte per mM creatinine. Nitrate-to-nitrite (NOx) ratios (R) were calculated for plasma (P; P_NOx_R) and urine (U; U_NOx_R) samples [47].

### 2.3. Statistical Analyses

Statistical analyses were performed using the statistical software package IBM^®^ SPSS^®^ Statistics for Windows, version 25.0 (IBM Corp., Armonk, NY, USA). Descriptive data were analyzed by the Chi-squared test or by Fisher’s exact test for groups smaller than five observations. The Kolmogorov–Smirnov test was used to test for normal distribution. Normally distributed data were analyzed using parametric tests (Student’s *t*-test, one-way ANOVA). Non-normally distributed data were analyzed using non-parametric tests (Mann–Whitney test, Kruskal–Wallis test). Bonferroni post hoc analysis was applied to ANOVA and Kruskal–Wallis test. Values of *p* < 0.05 were considered statistically significant. The data are presented as mean ± standard deviation (SD) or median (25th–75th interquartile range). Correlations were calculated as the Pearson correlation (rp) for normally distributed data and as the Spearman rank correlation (rs) for non-normally distributed data.

## 3. Results

### 3.1. Clinical Characterization of the CF Patients

Ten (14.3%; Table S1) CF patients had sufficient pancreatic function. Out of the 60 (85.7%; Table S1) pancreatic insufficient patients, eight patients showed liver involvement and two showed impairment in glucose metabolism. Overall, 11 (15.7%; Table S1) CF patients showed nutritional failure. BMIp ranged in the patients from 0.05 to 99.45 (average, 41.82; Table S1). Five (7.1%; Table S1) patients were classified as overweight according to Kromeyer-Hauschield et al. [35] and were included in the sufficiently nourished patients group. Sixteen (22.9%; Table S1) patients received oral or inhalative steroids. The Shwachman-Kulczycki score (Shwachmann-Score), rating the overall wellbeing of CF patients [48], ranked between 60 and 75; the Crispin-Norman Score, a radiological score describing lung damage in CF patients [49], ranged between 0 and 20. Forced expiratory volume in 1 s (FEV1%) is a measure for lung health and is most commonly reported in percent, comparing patients to a healthy age- and gender-matched population [50]. The minimum and maximum of FEV1% in the CF-patients were 36.1% and 126.1%, respectively (Table S1). Mid expiratory flow 25% (MEF25%) had a median at 59.5%. No signs of kidney impairment were seen in the CF patients based on plasma creatinine concentration and glomerular filtration rate (GFR) (mean GFR: 138 ± 20 mL/min).

### 3.2. The Arg/NO Pathway Status: Comparison of CF Patients with Healthy Controls

Plasma concentrations of Arg (*p* < 0.001), ADMA (*p* = 0.030), nitrate (*p* < 0.001), nitrite (*p* = 0.033) and the plasma Arg/ADMA ratio (*p* = 0.014) were significantly higher in the CF children and adolescents compared to the age-matched healthy controls (Table 1). There was no significant difference with respect to P_NOx_R between the groups (Table 1). Regarding urinary parameters, significantly higher DMA excretion rates (*p* < 0.001) were found in the CF patients compared to the healthy controls. The urinary DMA/ADMA ratio (*p* = 0.001), the nitrate excretion rate (*p* = 0.001) and the U_NOx_R (*p* = 0.003) were also higher in the CF patients compared to the healthy controls (Table 1).

### 3.3. The Arg/NO Pathway Status and the Involvement of Pancreas and Liver

Ten out of the 70 CF patients were pancreatic sufficient with no significant differences in body height (*p* = 0.47) or BMIp (*p* = 0.10) compared to pancreatic insufficient patients (Table S2). However, both prothrombin time (*p* = 0.031) and cholesterol (*p* = 0.005) were higher in these CF patients in comparison to the pancreatic insufficient CF patients (Table S2). None of the other clinical parameters differed between the pancreatic-sufficient and insufficient patients. The Arg/NO pathway status in the CF patients regarding the involvement of pancreas and liver is summarized in Table 2 and Table 3, respectively. Plasma citrulline was significantly higher in the pancreatic-insufficient CF patients than in the pancreatic-sufficient CF patients (*p* = 0.010). In comparison to healthy controls, pancreatic-sufficient CF patients had similar plasma Arg and ADMA concentrations compared to the healthy controls (Table 2), whereas pancreatic-insufficient CF patients had significantly higher plasma Arg and ADMA concentrations, i.e., Arg: insufficient vs. sufficient, *p* < 0.001; insufficient vs. control, *p* < 0.001; ADMA: insufficient vs. sufficient, *p* = 0.008; insufficient vs. control, *p* = 0.009 (Table 2). Pancreatic-insufficient CF patients had significantly higher plasma nitrate concentrations (*p* < 0.001) and urinary nitrate excretion rates (*p* < 0.001), as well as higher urinary DMA excretion rates (*p* < 0.001) and U_NOx_R values (*p* = 0.006) than healthy controls. The urinary DMA/ADMA ratio was higher in both, pancreatic sufficient (*p* = 0.008) and pancreatic insufficient (*p* = 0.018) CF patients when compared to healthy controls (Table 2). Furthermore, the plasma Arg/ADMA ratio was significantly higher in the pancreatic insufficient CF patients compared to the healthy controls (*p* = 0.014), but not in comparison to the pancreatic sufficient CF patients (*p* = 0.267). There were no differences between the pancreatic sufficient CF patients and the other two groups with respect to these parameters (Table 2).

CF patients with liver involvement had a significantly lower prothrombin time (*p* = 0.018), CHE (*p* = 0.006) and cholesterol (*p* = 0.024) values than CF patients without liver involvement; they tended to have lower urea concentrations (*p* = 0.058; Table S3). None of the other clinical parameters differed significantly between the eight CF patients with liver involvement and the 62 CF patients without liver involvement. Only the urinary DMA/ADMA ratio was significantly higher in the CF patients with liver involvement than in the controls (*p* = 0.005; Table 3). All the other observed differences depicted in Table 3 were significant only between the CF patients without liver involvement and the healthy controls.

### 3.4. The Arg/NO Pathway Status and the Nutritional Status of the CF Patients

Since nutritional failure was described to be involved in NO production, we split the CF patient group according to their nutritional status. Due to the missing complete data set on the nutritional status of the healthy controls, they could not be compared with CF patients. Eleven CF patients were classified as undernourished, with 9.1% being pancreatic sufficient and 45.5% being *P. aeruginosa* negative. CF patients with sufficient nourishment were found to have a higher plasma Arg/ADMA ratio (*p* = 0.021) than CF patients with insufficient nourishment (Table S4). The Arg plasma concentrations did not differ between the groups (*p* = 0.715), while the ADMA plasma concentrations tended (*p* = 0.060) to be higher (Table S4).

The Shwachmann-Score (*p* < 0.001), FEV1% (*p* = 0.001) and MEF25% (*p* = 0.038) were significantly higher in sufficiently nourished CF patients, while the Crispin-Norman Score (*p* = 0.003) was significantly lower (Table S5). CF patients with nutritional failure had significantly more frequent allergic bronchopulmonary aspergillosis (ABPA) than sufficiently nourished CF patients (*p* = 0.045). None of the other clinical parameters differed between the groups (Table S5).

As the Arg/NO pathway changes with age in children and adolescents [25], undernourished and sufficiently nourished pediatric patients were matched by age and gender (Table 4 and Table 5). In this matched subgroup, the plasma Arg/ADMA ratio remained significantly higher in the sufficiently nourished CF patients (*p* = 0.010), with the undernourished CF patients showing plasma Arg/ADMA ratios even lower than the healthy controls. Furthermore, ADMA excretion in the urine was significantly higher in the insufficiently nourished CF patients (*p* = 0.007). Furthermore, insufficiently nourished CF patients had significantly more severe respiratory impairment based on FEV1% (*p* = 0.011) and the Crispin-Norman Score (*p* = 0.005). Furthermore, the Shwachmann-Score was significantly lower in the CF patients with nutritional failure (*p* = 0.008). None of the other biochemical and disease parameters showed significant differences between the groups.

### 3.5. Correlation Analysis of Arg/NO Metabolites and Disease Parameters

No significant correlation was observed between Arg and age (*p* = 0.837), height percentile (*p* = 0.279), BMIp (*p* = 0.684), FEV1% *(p* = 0.315), the Shwachmann-Score (*p* = 0.554, or the Fischer-ratio (*p* = 0.319; data not shown). The Shwachmann-Score correlated positively both with the plasma Arg/ADMA ratio (Table S6) and the plasma nitrite concentration (Table S6). Additional significant correlations were found between the concentrations of the plasma and urinary members of the Arg/NO pathway in the CF patients. Especially P_NOx_R and U_NOx_R correlated with each other in the CF patients (*r* = 0.386, *p* = 0.001), but did not correlate with GFR (Table S6).

## 4. Discussion

The aim of the present systematic study was to quantitate the Arg/NO pathway in a large cohort of pediatric CF and in healthy children in plasma and urine with a special emphasis on nourishment and the involvement of the pancreas and liver, which are crucial factors in this disease. We measured the Arg and ADMA, the substrate and inhibitor of NO synthesis, the major NO metabolites nitrate and nitrite, and DMA, the main ADMA metabolite and measure of DDAH activity.

**Figure 1:**
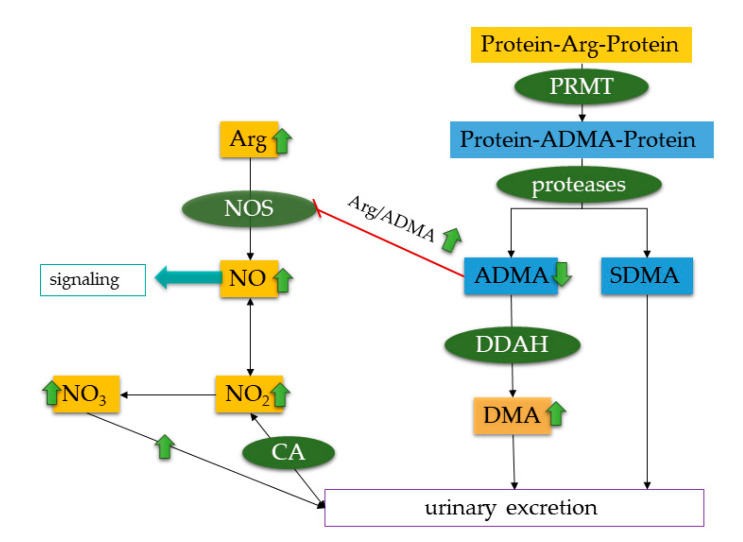
Simplified schematic of the L-arginine/nitric oxide pathway in healthy controls and changes in cystic fibrosis patients, including the low- and high-molecular-mass substrates, inhibitors, reaction products, enzymes. Green arrows indicate changes observed in the pediatric cystic fibrosis patients compared to healthy controls. Arg is metabolized by NOS, to build NO and citrulline (not shown). NO then may function as a signaling molecule and is quickly oxidized to form nitrite and nitrate, which are excreted in urine. The urinary excretion and reabsorption of nitrite is CA dependent. Arg in proteins might be methylated by PRMT to build ADMA or SDMA in proteins, which are released during proteolysis. ADMA is further metabolized by DDAH) to form DMA. DMA is the main metabolite of ADMA excreted in urine, but ADMA can be found in urine as well. ADMA and SDMA both are able to inhibit NOS activity. The Arg/ADMA ratio is a measure of NO synthesis capacity. Abbreviations: ADMA, asymmetric dimethylarginine; Arg, L-arginine; CA, carbonic anhydrase; DDAH, dimethylarginine dimethylaminohydrolase; NO, nitric oxide; NO2, nitrite; NO3, nitrate; NOS, nitric oxide synthase; PRMT, protein-arginine methyl transferase; SDMA; symmetric dimethylarginine. Double arrows indicate reactions might be reversable. The double-arrow on CA indicates that excretion and resorption of nitrite are CA-dependent. Green ellipses indicate enzymes involved in the metabolism. Rectangles indicate products in the Arg/NO metabolism, blue rectangles highlight products in the Arg/NO metabolism, which are considered atherosclerotic risk factors [51]. Yellow rectangles indicate all other Arg/NO metabolites. A red blunt-ended arrow indicates the inhibition of ADMA on NOS. A violet-marked rectangle indicates excretion, and a light blue rectangle indicates function of the molecule.

Our study reveals an altered Arg/NO pathway in CF patients compared to healthy age-matched controls. Arg, ADMA, nitrate and nitrite were elevated in the CF patients, and the metabolites of this pathway were excreted in urine accordingly in higher concentration than in healthy controls as shown in Figure 1. Pancreatic-sufficient patients had concentrations of the Arg/NO pathway comparable to those in healthy controls. Malnourished CF patients showed more severe lung impairment than well-nourished patients. The plasma Arg/ADMA ratio was lower in the malnourished patients. Therefore, the Arg/ADMA ratio might serve as an early marker of nutritional failure in pediatric CF (Figure 1). Additionally, the plasma Arg/ADMA ratio correlated with the Shwachmann-Score in all CF patients, indicating an association of higher Arg/ADMA ratio with better health in the CF patients group. These observations suggest differences in the Arg/NO pathway.

The Arg/NO pathway involves the NOS family, which consists of three isoforms, the constitutive and Ca^2+^-dependent endothelial NOS (eNOS) and neuronal NOS (nNOS) and the inducible, Ca^2+^-independent NOS (iNOS) [12,13]. The NOS activity is controlled by three guanidine (NG) methylated Arg analogues, i.e., NG-monomethyl-Arg (MMA), NG,NG-dimethylarginine (asymmetric dimethylarginine, ADMA), and NG,N’G-dimethylarginine (symmetric dimethylarginine, SDMA) all being endogenous inhibitors of NOS activity [30,52,53,54]. MMA, ADMA and SDMA are released during the proteolysis of certain NG-methylated proteins [52,54]. MMA is found in blood and urine at concentrations a 10th of the ADMA and SDMA concentrations [55,56]. ADMA and SDMA are considered atherosclerotic risk factors [51]. The protein precursors of MMA, ADMA and SDMA are produced and metabolized virtually in all organs, including the lungs. SDMA is mainly eliminated by the kidneys in its unchanged form. MMA is eliminated similarly to ADMA. The most important enzyme responsible for the metabolism of MMA and ADMA is dimethylarginine dimethylaminohydrolase (DDAH). This enzyme hydrolyzes MMA to monomethylamine and L-citrulline, whereas ADMA is hydrolyzed by DDAH to dimethylamine (DMA) and L-citrulline [17,18]. It is estimated that about 80% of daily endogenously produced ADMA is metabolized by DDAH and is excreted in the urine as DMA, with the remaining 20% being excreted in the urine as ADMA [19]. Urinary DMA is therefore a useful measure of whole-body ADMA synthesis [19].

Nitrite and nitrate are major metabolites of NO. They circulate in the blood and are excreted mainly in the urine. Circulating nitrite and nitrate are useful indicators of systemic NO synthesis, while urinary nitrate rather represents a measure of whole-body NO synthesis. It is worth mentioning that a considerable fraction of circulating and urinary nitrite and nitrate derives from nutrition [15]. Furthermore, NO can also be produced from inorganic nitrite in various compartments of the body under certain conditions, such as hypoxia or upon bacterial nitrate reductase-catalyzed reduction of nitrate to nitrite in the gut and mouth flora, independent of the Arg/NO pathway [38,57].

Our finding of higher plasma Arg concentration in pediatric patients with CF is in line with Engelen et al. [29]. Yet, in adult CF patients, lower Arg plasma concentrations were measured during pulmonary exacerbation that were normalized after 14 days of antibiotic treatment [28]. Interestingly, CF patients studied by Engelen et al. [29] were tested after antibiotic treatment only, while none of our CF patients had received antibiotic treatment prior to examination. Stable CF patients seem to show elevated plasma Arg levels.

The plasma Arg/ADMA ratio is considered a measure of NO synthesis capacity [26,30,31]. In our pediatric CF patients, the plasma Arg/ADMA ratio was higher than in our controls, albeit of a relatively low degree of 9%. NOS activity depends on the local relative bioavailability of Arg and ADMA, i.e., on the cellular Arg uptake. Arg and L-ornithine, which is produced from Arg by the catalytic action of arginase, compete on the same amino acid transporter [28,58]. Therefore, rising L-ornithine concentrations, for instance due to elevated arginase activity, could lead to lower cellular Arg concentrations, which may not necessarily correlate with extracellular Arg concentrations. In plasma of our CF patients, we measured systemic Arg concentrations, which should have been sufficient to ensure high Arg bioavailability in NO-producing cells. This is supported by the observation of higher plasma and urinary nitrate concentrations in the CF patients compared to healthy age-matched children, both in plasma and in urine. Hence, in our pediatric CF patients, NOS activity seems not to be impaired.

### 4.1. Potential Roles of Liver, Pancreas and Kidney in Pediatric Cystic Fibrosis

ADMA is produced through the post-translational dimethylation of arginine residues in certain proteins by the catalytic action of protein-arginine methyltransferase (PRMT). PRMT and DDAH, which hydrolyzes ADMA to DMA and L-citrulline [54], are widely distributed in human organism including lung, liver, kidneys and pancreas [59]. In our CF patients, we observed higher PRMT activity, as measured by higher ADMA concentrations in plasma and urine, and higher DDAH activity, as measured by the DMA/ADMA ratio in urine, compared to the healthy children. The urinary DMA/ADMA ratio is a measure of the whole-body balance of PRMT and DDAH activity [44]. The urinary DMA/ADMA ratio was by about 21% higher in the CF patients compared to the healthy controls, indicating enhanced activity of DDAH and PRMT. In adults, the urinary DMA/ADMA ratio was found to be lower in patients suffering from liver disease compared to healthy subjects [44]. These observations may suggest that liver disease only had little contribution to whole-body DDAH/PRMT balance. The liver impairment of our CF patients was relatively mild, as suggested by the slightly decreased CHE activity and albumin concentration compared to the healthy CF patients. CF patients with liver involvement had 23% lower DMA/ADMA ratios than CF patients with no liver involvement. CF patients with pancreas insufficiency had by 9% lower DMA/ADMA ratios than CF patients with pancreas sufficiency. These diametric effects suggest that the liver may have a relatively stronger effect on the whole-body DDAH/PRMT state in pediatric CF. The lung has been identified as a major source of ADMA, i.e., of PRMT activity [59]. This observation and our present results suggest that PRMT activity is considerably enhanced in the lung of our CF patients, presumably due to chronic inflammation, and contributes to circulating and urinary ADMA.

Plasma creatinine concentration and GFR did not indicate kidney impairment in our CF patients. P_NOx_R and U_NOx_R correlated with each other in the CF patients, but they did not correlate with GFR. This observation may suggest that both the plasma and the urinary concentrations of nitrate and nitrite are primarily dependent upon the reabsorption of nitrite by carbonic anhydrase (CA) in the proximal tubule of the nephron [47] (Figure 1). By using the drug acetazolamide, a strong inhibitor of CA activity, early work found that CA is involved in CF [60,61,62]. Acetazolamide administration to cats was found to reduce bicarbonate concentration and to increase the concentration of chloride in the sweat [62]. In the meantime, there is strong evidence that CFTR and CA are co-expressed in various cells and organs, such as pancreatic and bile duct epithelial cells, including several nephron segments of mammalian kidney, mediate the bicarbonate transport, and are involved in the transport of other anions and cations, including Na^+^ and K^+^ by other transporters [63,64,65,66,67,68]. Yet, whether renal CFTR plays a role in the excretion and/or reabsorption of nitrite and nitrate is not known. In our CF patients, we found higher (by 33%) U_NOx_R values than in the healthy controls, presumably due to a higher excretion rate of nitrate (by 28%) and a lower (by 17%), albeit insignificant, urinary nitrite excretion. The U_NOx_R values measured in the CF children are closely comparable with those measured in type I diabetic children [47]. CF children with insufficient pancreas function had higher (by 11%) U_NOx_R values than CF children with sufficient pancreas, while CF children with liver involvement had lower (by 13%) U_NOx_R values than CF children with insufficient pancreas function. These observations may suggest the opposite effects of the pancreas and liver on the renal excretion of nitrite and nitrate in pediatric CF. The urinary excretion rates of nitrite do not give reason for bacterial infection (e.g., nitrate reductase) in the urogenital tract of the children.

In our pancreatic insufficient CF patients, elevated Arg, ADMA and nitrate in plasma were observed. Additionally, Krantz et al. observed higher calculated alveolar NO in pancreatic insufficient CF patients, compared to pancreatic sufficient CF patients [69]. Taken together this might indicate escalated inflammation and heightened NO production in pancreatic insufficient CF patients. Plasma Arg concentrations comparable to our CF patients were also observed in pediatric diabetes type I patients under long-term insulin treatment [23]. Unlike CF patients, diabetes type I patients showed no elevation in plasma ADMA concentrations and significantly lower plasma nitrite concentrations compared to healthy controls [23]. The elevation of Arg levels results in enhanced insulin excretion [70]. With a large amount of β-cells being lost in CF patients, individual cells need to produce larger amounts of insulin than in the healthy pancreas [71,72]. The involvement of NOS activity in β-cell damage during diabetes type I has been described. The elevation of NO production during inflammation in diabetes type I pancreas leads to the elevated apoptosis of β-cells [73]. Additionally it has been described for β-cells themselves to over-produce NO in diabetes type II, while the low production of NO results in insulin excretion [71,74]. Elevated Arg and nitrate in the plasma of our CF patients might be a measure of elevated insulin excretion, while the pancreas itself is inflamed, especially since it still is not fully understood why the number of CF patients’ islets is reduced [75]. As CFTR is not involved in insulin secretion, islets of CF patients are not inherently constricted. Rather, the loss of function of the endocrine pancreas stems from several factors, such as malnutrition and malabsorption, inflammation and multi-organ failure in CF [72]. Therefore, the missing activation of the Arg/NO metabolism in pancreatic-sufficient CF patients might reflect a healthy pancreatic situation without inflammation or counteracting reduced insulin production due to β-cell loss.

### 4.2. Potential ADMA-Related Cardiovascular Risk in Pediatric Cystic Fibrosis

In adults, a rise of about 11–46% in circulating ADMA concentrations is related to severe outcomes in coronary artery disease (CAD) and death [76,77]. In the last decades, up to 50% of CF patients reached adulthood, most likely due to improved care [78]. Since CF patients are getting older, the consequences of chronic disease and aging become a more prevalent issue in the aging CF community. Chronic inflammation and pulmonary obstruction as well as a high-fat diet contribute to increased cardiovascular risk in aging CF patients [79]. Even today, it is still unclear if these elevated risk factors are leading to CAD and impaired vascular function in CF [76,79,80,81]. While Vizzardi et al. [80] found a higher frequency of endothelial dysfunction in adult CF patients (mean age, 24 years), Skolnik et al. [79] did not find any atherosclerotic plaque in their adult CF patients (mean age, 47 years). Chronic inflammation might reduce NO bioavailability in CF patients, thus increasing cardiovascular risk [80]. The CF children (age, 5–17 years) of our study had, by 8% higher plasma ADMA concentrations than the age-matched healthy children. The 60 CF children with pancreas insufficiency had, by 17%, higher plasma ADMA concentrations than the 10 CF children with pancreas sufficiency. The eight CF children with liver involvement had, by 3%, lower plasma ADMA concentrations than the 62 CF children with no liver involvement. With respect to nourishment, the 11 CF patients with nutritional failure had, by 10%, higher plasma ADMA concentrations than the 11 CF patients with sufficient nourishment.

Despite higher plasma ADMA concentrations, we observed noticeably higher NO production in the CF children compared to the healthy control. Yet, because circulating and urinary nitrite and nitrate originate from eNOS, nNOS and iNOS and in part from nutrition, which has not been controlled in our study, it is actually impossible to specify the contribution of each origin to NO production [38]. Furthermore, neither circulating nor excretory nitrite and nitrate provide dependable information on the bioavailability on NO at its site of formation. Whether circulating ADMA represents a cardiovascular risk in children, is still unknown. Our study cannot predict future outcomes of cardiovascular disease risk. Labombarda et al. [81] identified myocardial disease in CF patients as a common diagnosis, probably provoked through CFTR involvement during contraction of the myocardium. Free ADMA and ADMA-containing proteins are assumed to exert additional not yet fully understood effects [82]. There is increasing evidence that cardiac sodium channels are post-translationally dimethylated on certain arginine residues and that this modification is associated with altered function [83,84,85].

As these abnormalities lead to abnormalities in systolic and diastolic functions, further research of cardiovascular risk joint with myocardial disease might be of interest in the aging CF population. A longtime observation of the Arg/NO pathway, including ADMA, might help understand changes in epithelial and endothelial functions in CF patients.

### 4.3. External Sources of Arg, ADMA and DMA

The daily dietary intake of Arg is estimated to be 2–7 g; Arg is well absorbed and results in plasma peak levels about 2 h after oral admission [11]. To the best of our knowledge no such data is available for ADMA. The oral administration of L-citrulline was also shown to result in increasing plasma Arg concentrations, even outperforming Arg supplementation itself [11,86]. Rougé et al. observed an elevation of citrulline by about 500% after the supplementation of citrulline in adults and an increase of 100% of Arg [86]. The higher plasma citrulline concentrations in our pancreatic insufficient CF patients could have led to the higher Arg plasma concentrations in these patients. The intake of Arg-rich proteins may have contributed to higher blood Arg levels in CF patients [87]. Intake of higher amounts of Arg or citrulline as part of standardized pharmaceutical therapy, such as gelatin (100 g gelatin contain 7.5 g Arg [88]), is likely to lead to higher Arg and citrulline concentrations in the blood. Supplementation of 9.96 g/d of Arg for six months increased plasma Arg concentration in 31 elderly CAD patients from 67.7 µM to 94.7 µM; the same dose for 3 months in 20 peripheral arterial occlusive disease (PAOD) patients resulted in a mean increase in plasma Arg concentration from 56.5 to 80 µM [89]. These elevations were associated with marginal increases in plasma ADMA in the CAD and PAOD patients [89]. The 1.4-fold increase is comparable to the 1.2-fold higher Arg plasma concentration we observed in our CF children. The loss of pancreatic enzymes might occur in CF patients with pancreatic insufficiency. Yet, data on the fate of pancreatic enzymes such as amylase and lipase in the small intestine are scarce and controversial [90,91]. Elevated Arg plasma concentrations in our pancreatic insufficient CF patients either could be a result of a reduced loss of Arg through pancreatic enzymes or of an elevated uptake of Arg through the substitution of pancreatic enzymes.

### 4.4. Arg/NO Metabolism in Nutritional Failure

ADMA is built by the methylation of Arg residues in proteins and is released by proteolysis [59]. Consequently, higher ADMA plasma (although not significant) and urinary concentrations in our CF patients with nutritional failure could stem from a catabolic state, as Engelen et al. [29] published similar findings. Since methylarginine metabolism in the lungs contributes significantly to circulating ADMA [59] and undernourished CF patients show increased respiratory impairment, ADMA production in the lungs of these CF patients might as well be elevated. As the synthesis of NO not only is modulated by Arg availability and ADMA inhibition, further studies on the complex interactions and modulations of NO synthesis would be of interest. In adults, insulin like growth factor-1 (IGF-1) is diminished significantly after five days of fasting and is only sufficiently restored during refeeding with sufficient energy and protein intake [92], while long-term calorie restriction with adequate protein intake did not influence IGF-1 [93]. The same results were observed in anorexic adolescents and young adults and children suffering from malnutrition [94,95]. It has been observed that IGF-1, as well as insulin, stimulates NO production in endothelial cells [96]. In CF mice, IGF-1 reduction was seen in late adolescence and reduced weight and length occurred independent of gastrointestinal CF issues [97]. As chronic inflammation in CF patients could lower IGF-1 concentration, NO production could be influenced as well as the possibility that respiratory impairment might rise as IGF-1 levels decrease [98]. In 56 community-dwelling elderly, a two-week supplementation of energy and protein resulted in elevated IGF-1 concentration and the lowering of inflammation markers interleukin 6 and tumor necrosis-factor α [99]. In pediatric CF patients, growth hormone treatment without a prior growth hormone deficit lead to the improvement of growth, nutritional status, protein retention and IGF-1 levels [100,101]. Interconnection of nutritional failure and IGF-1 might therefore also influence inflammation. Imbalances of structures activating NOS might be influenced by reduced Arg/ADMA ratio in CF patients with nutritional failure as we observed. Langen et al. observed a positive correlation of IGF-1 and homoarginine/ADMA ratio in children of short stature without growth hormone deficiency. Additionally, they observed a positive correlation of IGF-1 with homoarginine as well as homoarginine/ADMA ratio in children of short stature with growth hormone deficiency [102], suggesting coupling of NO metabolism and IGF-1. The coupling of NO and IGF-1 metabolic and signaling pathways might highlight early markers, such as the inhibition of NOS measured as a lower Arg/ADMA ratio in our CF patients, for growth hormone deficiencies and nutritional failure.

Patients with anorexia nervosa and very low body weight show complications in the cardiovascular system, especially pericardial effusion, which is related to very low body weight and reversed with weight gain [103,104]. According to the loss of cardiac muscle, anorexia nervosa patients present lower blood pressure, which resolves in institutionalization [105,106,107]. Furthermore, elevated FENO was observed, while Rodrigue Pereira et al. reported diminished Arg plasma concentration in anorexic patients [33,34]. Vignini et al. [108] observed reduced Arg in anorexic patients’ plasma with elevated NO production in platelets, which normalized after two weeks of Arg supplementation. As we observed changes in the Arg metabolism in very-low-weight CF patients, observations of the Arg/NO metabolism in anorexia nervosa patients might result in findings of early intervention markers in anorexia patients as well. Especially since severe complications in anorexia result in sudden death due to cardiovascular changes, early intervention markers might be useful [105,106,107,109]. Imbalances in NO production in insufficiently nourished CF patients and anorexia nervosa patients might further facilitate insight into the complex regulation of Arg/NO metabolism.

As Arg/ADMA ratio was significantly reduced in insufficiently nourished CF patients, without significant differences in Arg or ADMA plasma levels, longitudinal analysis of this ratio might uncover early alterations in metabolism hinting to nutritional failure even before BMI falls below 10th percentile. Furthermore, increased urinary ADMA excretion might be considered a measure against rising ADMA plasma levels and should therefore be included into longitudinal observations. Especially as small ADMA plasma elevations are related to severe outcomes in adults [76], elevated urinary excretion in pediatric CF patients might serve as an early marker for metabolic imbalances.

### 4.5. Study Limitations

Most of the pediatric CF patients studied here showed mild disease with only two out of 70 showing impaired glucose metabolism. Lung function, measured as FEV1%, ranged between 126% and 36% (90% ± 19% (mean ± standard deviation)), only two CF patients showed FEV1% below 50%. Hence, the activation of the Arg/NO metabolism in the CF patients cannot be associated with a decline in lung function. Longitudinally designed studies could shed light on changes in Arg/NO metabolism through disease progression and the aging of the CF patients. As the BMI data of controls were missing, no comparison of CF and controls was conducted regarding malnutrition. One major limitation to consider is that CF patients were not fasted overnight and were not required to refrain from foods rich in citrulline, Arg, nitrate and nitrite prior to examination, although they were asked to avoid fresh or canned fish, which may contribute to urinary DMA concentration [110]. As discussed above, citrulline ingestion leads to an elevation in Arg and citrulline plasma concentration and therefore might have influenced Arg and citrulline concentration. However, standard deviations in Arg and citrulline concentration were comparable in all groups, indicating the non-fasted status and differences of time spent since last meal until blood drawing might not be of interest. Furthermore, not all parameters were analyzed in the control group. Additionally, we cannot draw conclusions about the actual Arg/NO content in individual tissues, e.g., lung, airway, neuronal or pancreatic tissue.

## 5. Conclusions

Pediatric CF patients with mild disease have higher plasma and urinary concentrations of Arg and of the NO metabolites nitrate and nitrite compared to healthy controls indicating both sufficient Arg availability from endogenous and exogenous sources and activated Arg/NO pathway. Pancreatic-sufficient CF patients and healthy controls have a similar Arg/NO metabolism, suggesting Arg/NO modulation as an interesting therapeutic target in pancreatic insufficient patients. The plasma Arg/ADMA ratio was lower in the malnourished CF patients, suggesting lower NO synthesis capacity in these patients, which might be a useful marker for nutritional failure. Pancreas and liver function seem to have opposite effects on the renal excretion of nitrite and nitrate. The contribution of renal CA and CFTR to the excretion/reabsorption of nitrite and nitrate in pediatric CF is elusive and warrants further investigations.

As the CF population is continuing to have higher life expectancy, cardiovascular issues might arise. The influence of the Arg/NO pathway and CFTR deficiency on myocardial disease and cardiovascular risk could become a field of interest in future research on CF. Here, attention on changes in Arg and ADMA might be of interest in disease progression.

## Figures and Tables

**Table 1:** Altered L-arginine (Arg)/nitric oxide (NO) status in the plasma (P) and urine (U) of children and adolescents with cystic fibrosis (CF) and of healthy controls.

	CF Patients	Healthy Controls	*p*
Number of subjects (*n*)	70	78	
Age (years)	11.8 (8.25–14.0)	11.3 (8.19–13.2)	0.450
Male [*n* (%)]	30 (42.9)	39 (50)	0.384
**Arg** (P) **(µM)**	**90.3 ± 20.4**	**75.6 ± 18.6**	**<0.001**
Citrulline (**P**) (µM)	36 (29–42)	not measured	-
ADMA (P) (µM)	0.62 ± 0.12	0.57 ± 0.11	0.030
**Arg/ADMA ratio (P)**	**148 ± 27.9**	**135 ± 33.7**	**0.014**
**Nitrate** (P) **(µM)**	**43.3 (37.4–51.9)**	**33.1 (27.1–42.8)**	**<0.001**
**Nitrite** (P) **(µM)**	**2.07 (1.86–2.28** **)**	**1.95 (0.83–2.33)**	**0.033**
P_NOx_R	20.9 (17.3–24.3)	22.8 (14.0–41.9)	0.518
ADMA (U) (µM/mM creatinine)	8.17 ± 3.21	7.21 ± 3.13	0.088
**DMA (U) (µM/mM creatinine)**	**57.9 (47.0–70.4)**	**40.7 (25.9–56.0)**	**<0.001**
**DMA/ADMA (U)**	**7.62 (6.40–8.54)**	**6.05 (4.12–9.29)**	**0.001**
**Nitrate (U) (µM/mM creatinine)**	**159 (113–221)**	**115 (80.2–156)**	**0.001**
Nitrite (U) (µM/mM creatinine)	0.20 (0.13–0.35)	0.24 (0.16–0.46)	0.478
**U_NOx_R**	**746 (498–1022)**	**501 (285–864)**	**0.003**

Abbreviations. Arg, arginine; ADMA, asymmetric dimethylarginine; DMA, dimethylamine; P, plasma; U, urine; P_NOx_R, plasma nitrate/nitrite ratio; U_NOx_R, urinary nitrate/nitrite ratio. The data are reported as median (25–75 percentile) (non-normal distribution) or as mean ± standard deviation (normal distribution). Significant results are marked in bold.

**Table 2:** Arg/NO status in plasma (P) and urine (U) in the patients with cystic fibrosis (CF) with regard to their pancreatic sufficiency and healthy controls.

	CF Patients		Healthy Controls	*p*
	Insufficient pancreas	Sufficient pancreas		
Number of subjects (*n*)	60	10	78	-
Age (years)	11.1 [8.23–13.7]	13.2 [9.65–15.4]	11.3 [8.19–13.2]	0.317
Male (*n* (%))	25 (41.7)	5 (50.0)	39 (50.0)	0.636^+^
**Arg (** **P) (µM)**	**94.1 ± 18.8**	**68.3 ± 15.5**	**75.6 ± 18.6**	**<0.001**
**Citrulline (P) (µM)**	**37.0 [31.0–44.0]**	**28.5 (23.3–36.0)**	**not measured**	**0.010**
**ADMA (P) (µM)**	**0.63 ± 0.12**	**0.52 ± 0.06**	**0.57 ± 0.11**	**0.001**
**Arg/ADMA ratio (*P*)**	**150 ± 27.3**	**132 ± 27.8**	**134 ± 33.7**	**0.012**
**Nitrate (P) (µM)**	**44.1 (38.5–52.2)**	**38.4 (26.7–45.7)**	**33.1 (27.1–42.8)**	**<0.001**
Nitrite (**P**) (µM)	2.06 (1.86–2.27)	2.07 (1.94–2.42)	1.95 (0.83–2.33)	0.100
P_NOx_R	21.5 (18.3–25.3)	15.8 (12.5–22.0)	22.8 (14.0–41.9)	0.177
ADMA (U) (µM/mM creatinine)	8.26 ± 2.42	7.64 ± 6.27	7.21 ± 3.13	0.201
**DMA (U)** **(µM/mM creatinine)**	**57.9 (48.4–70.5)**	**52.7 (43.9–65.1)**	**40.7 (25.9–56.0)**	**<0.001**
**DMA/ADMA (U)**	**7.33 (6.37–8.31)**	**8.06 (7.67–11.1)**	**6.05 (4.12–9.29)**	**0.002**
**Nitrate (U)** **(µM/mM creatinine)**	**167 (119–239)**	**102 (73.3–184)**	**115 (80.2–156)**	**<0.001**
Nitrite (U) (µM/mM creatinine)	0.22 (0.15–0.35)	0.13 (0.11–0.34)	0.24 (0.16–0.46)	0.338
**U_NOx_R**	**752 (511–1025)**	**666 (391–864)**	**501 (285–864)**	**0.009**

Abbreviations. Arg, arginine; ADMA, asymmetric dimethylarginine; DMA, dimethylamine; P, plasma; U, urine; P_NOx_R, plasma nitrate/nitrite ratio; U_NOx_R, urinary nitrate/nitrite ratio. The data are reported as median (25–75 percentile] (non-normal distribution) or as mean ± standard deviation (normal distribution). The results of the Bonferroni post-hoc analysis are presented in the text. ^+^ Fisher’s exact test. Significant results are marked in bold.

**Table 3:** Arg/NO status in plasma (P) and urine (U) in the patients with cystic fibrosis (CF) with regard to their liver health and healthy controls.

	CF Patients		Healthy Control	*p*
	**Liver involvement**	**No liver involvement**		
Number of subjects (*n*)	8	62	78	-
Age years	12.0 (7.86–17.1)	11.8 (8.25–14.0)	11.3 (8.19–13.2)	0.611
Male (*n* (%))	3 (37.5)	27 (43.5)	39 (50.0)	0.707^+^
**Arg (** **P) (µM)**	**83.3 ± 18.8**	**91.2 ± 20.6**	**75.6 ± 18.6**	**<0.001**
Citrulline (P) (µM)	36.5 (28.3–38.5)	36.0 (29.0–43.5)	not measured	0.619
ADMA (P) (µM)	0.60 ± 0.16	0.62 ± 0.12	0.57 ± 0.11	0.088
Arg/ADMA (**P**)	141 ± 16.4	149 ± 29.1	135 ± 33.7	0.040
**Nitrate (P) (µM)**	**39.0 (35.4–40.9)**	**44.4 (37.8–52.8)**	**33.1 (27.1–42.8)**	**<0.001**
Nitrite (P) (µM)	2.06 (1.63–2.37)	2.07 (1.86–2.27)	1.95 (0.83–2.33)	0.100
*P*_NOx_R	18.3 (16.3–21.8)	21.4 (17.6–25.1)	22.8 (14.0–41.9)	0.531
ADMA (U) (µM/mM creatinine)	7.14 ± 2.76	8.31 ± 3.26	7.21 ± 3.13	0.146
**DMA (U)** **(µM/mM creatinine)**	**57.3 (41.5–87.8)**	**58.2 (47.7–70.2)**	**40.7 (25.9–56.0)**	**<0.001**
**DMA/ADMA (U)**	**9.64 (6.93–12.2)**	**7.41 (6.36–8.16)**	**6.05 (4.12–9.29)**	**0.001**
**Nitrate (U)** **(µM/mM creatinine)**	**110 (90.0–229)**	**167 (120–221)**	**115 (80.2–156)**	**0.002**
Nitrite (U) (µM/mM creatinine)	0.17 (0.09–0.81)	0.20 (0.14–0.35)	0.24 (0.16–0.46)	0.707
**U_NOx_R**	**652 (304–986]**	**746 (576–1026]**	**501 (285–864]**	**0.007**

Abbreviations. Arg, arginine; ADMA, asymmetric dimethylarginine; DMA, dimethylamine; P, plasma; U, urine; P_NOx_R, plasma nitrate/nitrite ratio; U_NOx_R, urinary nitrate/nitrite ratio. The data are reported as median (25–75 percentile) (non-normal distribution) or as mean ± standard deviation (normal distribution). The results of the Bonferroni post-hoc analysis are presented in the text. ^+^ Fisher’s exact test. Significant results are marked in bold.

**Table 4:** Age- and gender-matched comparison of cystic fibrosis (CF) patients with nutritional failure with CF patients with sufficient nourishment with regard to the Arg/NO pathway status in plasma (P) and urine (U).

	CF Patients		*p*
	**Nutritional Failure ^a^**	**Sufficient Nourishment** **^b^**	
Number of subjects (*n*)	11	11	-
Age (years)	13.2 (10.2–14.2)	13.1 (10.1–14.7)	0.949
Male (*n* (%))	3 (27.3)	3 (27.3)	1.000
Systolic blood pressure (mmHg)	115 (108–116)	114 (112–120)	0.606
Diastolic blood pressure (mmHg)	62 (60–72)	70 (60–75)	0.365
Arg (P) (µM)	88.2 ± 16.8	95.2 ± 18.3	0.367
Citrulline (P) (µM)	33.3 ± 10.2	37.2 ± 9.26	0.358
ADMA (P) (µM)	0.68 ± 0.11	0.61 ± 0.12	0.169
**Arg/ADMA (** **P)**	**130 ± 19.1**	**158 ± 26.5**	**0.010**
Nitrate (P) (µM)	50.4 ± 16.6	44.1 ± 12.4	0.322
Nitrite (P) (µM)	2.03 (1.85–2.34)	1.91 (1.81–2.70)	0.748
P_NOx_R	25.1 ± 9.07	20.6 ± 6.25	0.191
**ADMA (U)** **(µM/mM creatinine)**	**8.93 (7.12–10.3)**	**6.53 (5.40–7.58)**	**0.007**
DMA (U) (µM/mM creatinine)	56.5 (52.3–65.6)	46.6 (43.5–70.9)	0.151
DMA/ADMA	7.31 (6.05–7.79)	7.84 (6.42–10.3)	0.193
Nitrate (U) (µM/mM creatinine)	203 ± 105	142 ± 57.2	0.105
Nitrite (U) (µM/mM creatinine)	0.24 ± 0.10	0.18 ± 0.08	0.120
U_NOx_R	912 ± 404	855 ± 277	0.701

^a^ Nutritional failure is defined as a BMI percentile < 10; ^b^ sufficient nourishment is defined as a BMI percentile ≥ 10. Abbreviations. Arg, arginine; ADMA, asymmetric dimethylarginine; DMA, dimethylamine; P, plasma; U, urine; P_NOx_R, plasma nitrate/nitrite ratio; U_NOx_R, urinary nitrate/nitrite ratio. The data are reported as median (25–75 percentile) (non-normal distribution) or as mean ± standard deviation (normal distribution). Significant results are marked in bold.

**Table 5:** Age- and gender-matched comparison of cystic fibrosis (CF) patients with nutritional failure and sufficiently nourished CF patients with regard to anthropometric and clinical parameters.

	CF Patients		*p*
	**Nutritional Failure ^a^**	**Sufficient Nourishment ^b^**	
Number of subjects (*n*)	11	11	-
Height (percentile)	35.0 ± 27.2	39.1 ± 21.7	0.699
**BMIp (percentile)**	**3.56 (1.25–6.75)**	**45.9 (28.0–68.2)**	**<0.001**
**Shwachmann-Score**	**70 (65–75)**	**75 (75–75)**	**0.008**
**FEV1%**	**73.0 ± 22.2**	**96.2 ± 16.3**	**0.011**
MEF25%	43.3 ± 27.6	74.1 ± 53.4	0.105
**Crispin-Norman-Score**	**10.4 ± 5.39**	**4.55 ± 2.94**	**0.005**
FENO (ppb)	8.20 (4.70–15.7)	10.2 (7.08–13.2)	0.765
Fischer-Quotient	3.50 ± 0.85	3.27 ± 0.43	0.432
Prothrombin time (%)	81.8 ± 9.38	81.2 ± 11.6	0.889
Cholesterol (mM)	3.39 ± 0.57	3.62 ± 0.91	0.538
Triglycerides (mM)	1.05 (0.77–1.37)	1.36 (0.92–2.12)	0.203
Urea (mM)	3.95 ± 0.79	4.07 ± 1.19	0.787
**GFR (mL/min)**	**145 ± 11.4**	**129 ± 17.6**	**0.020**
Pancreas sufficiency (*n* (%))	1 (9.1)	2 (18.2)	1.00^+^
Liver involvement (*n* (%))	1 (9.1)	2 (18.2)	1.00^+^
*P. aeruginosa* negative (*n* (%))	5 (45.5)	8 (72.2)	0.387^+^
ABPA (*n* (%))	3 (27.3)	1 (9.1)	0.586^+^
Acute infect (*n* (%))	6 (54.5)	5 (45.5)	1.00^+^
Steroid treatment (*n* (%))	5 (45.5)	2 (18.2)	0.361^+^

^a^ Nutritional failure is defined as a BMI percentile < 10; ^b^ sufficient nourishment is defined as a BMI percentile ≥ 10. Abbreviations. BMIp, BMI percentile; FEV1%, Forced expiratory volume in 1 s; MEF25%, mid expiratory flow 25%; GRF, glomerular filtration rate; ABPA, allergic bronchopulmonary aspergillosis. The data are reported as median (25–75 percentile) (non-normal distribution) or as mean ± standard deviation (normal distribution). ^+^ Fisher’s exact test. Significant results are marked in bold.

## Supplementary Materials

The following are available online at https://www.mdpi.com/2077-0383/9/6/2012/s1, Table S1 Anthropometric and clinical characteristics of cystic fibrosis (CF) patients; Table S2 Anthropometric and clinical characteristics of cystic fibrosis (CF) patients according to their pancreas sufficiency; Table S3 Anthropometric and clinical characteristics of cystic fibrosis (CF) patients according to the involvement of liver function; Table S4 Anthropometric characteristics and biochemical parameters in plasma (P) and urine (U) of the cystic fibrosis patients according to the nutritional status; Table S5 Anthropometric and clinical characteristics of the cystic fibrosis (CF) patients according to their nutritional status; Table S6 Summary of the results of Spearman correlation analyses between members of the Arg/NO pathway in plasma (P) and urine (U) in the cystic fibrosis patients of the study.

## References

[1] Naehrig S., Chao C.-M., Naehrlich L. (2017). Cystic Fibrosis. Dtsch. Arztebl. Int..

[2] Fabris L., Fiorotto R., Spirli C., Cadamuro M., Mariotti V., Perugorria M.J., Banales J.M., Strazzabosco M. (2019). Pathobiology of inherited biliary diseases: A roadmap to understand acquired liver diseases. Nat. Rev. Gastroenterol. Hepatol..

[3] Cohen T.S., Prince A. (2012). Cystic fibrosis: A mucosal immunodeficiency syndrome. Nat. Med..

[4] Castellani C., Assael B.M. (2017). Cystic fibrosis: A clinical view. Cell. Mol. Life Sci..

[5] Wilschanski M., Novak I. (2013). The Cystic Fibrosis of Exocrine Pancreas. Cold Spring Harb. Perspect. Med..

[6] Turck D., Braegger C.P., Colombo C., Declercq D., Morton A., Pancheva R., Robberecht E., Stern M., Strandvik B., Wolfe S. (2016). ESPEN-ESPGHAN-ECFS guidelines on nutrition care for infants, children, and adults with cystic fibrosis. Clin. Nutr..

[7] Gibson-Corley K.N., Meyerholz D.K., Engelhardt J.F. (2016). Pancreatic pathophysiology in cystic fibrosis. J. Pathol..

[8] Flass T., Narkewicz M.R. (2013). Cirrhosis and other liver disease in cystic fibrosis. J. Cyst. Fibros..

[9] Hollander F.M., de Roos N.M., Heijerman H.G.M. (2017). The optimal approach to nutrition and cystic fibrosis: Latest evidence and recommendations. Curr. Opin. Pulm. Med..

[10] Wu G., Bazer F.W., Davis T.A., Kim S.W., Li P., Marc Rhoads J., Carey Satterfield M., Smith S.B., Spencer T.E., Yin Y. (2009). Arginine metabolism and nutrition in growth, health and disease. Amino Acids.

[11] Morris C.R., Hamilton-Reeves J., Martindale R.G., Sarav M., Ochoa Gautier J.B. (2017). Acquired Amino Acid Deficiencies: A Focus on Arginine and Glutamine. Nutr. Clin. Pract..

[12] Lundberg J.O., Gladwin M.T., Weitzberg E. (2015). Strategies to increase nitric oxide signalling in cardiovascular disease. Nat. Rev. Drug Discov..

[13] Chachlaki K., Garthwaite J., Prevot V. (2017). The gentle art of saying NO: How nitric oxide gets things done in the hypothalamus. Nat. Rev. Endocrinol..

[14] Lei J., Vodovotz Y., Tzeng E., Billiar T.R. (2013). Nitric oxide, a protective molecule in the cardiovascular system. Nitric Oxide.

[15] Webb A.J., Patel N., Loukogeorgakis S., Okorie M., Aboud Z., Misra S., Rashid R., Miall P., Deanfield J., Benjamin N. (2008). Acute Blood Pressure Lowering, Vasoprotective, and Antiplatelet Properties of Dietary Nitrate via Bioconversion to Nitrite. Hypertension.

[16] Leone A., Moncada S., Vallance P., Calver A., Collier J. (1992). Accumulation of an endogenous inhibitor of nitric oxide synthesis in chronic renal failure. Lancet.

[17] Ogawa T., Kimoto M., Sasaoka K. (1989). Purification and properties of a new enzyme, NG,NG-dimethylarginine dimethylaminohydrolase, from rat kidney. J. Biol. Chem..

[18] Ogawa T., Kimoto M., Sasaoka K. (1987). Occurrence of a new enzyme catalyzing the direct conversion of NG,NG-dimethyl-L-arginine to L-citrulline in rats. Biochem. Biophys. Res. Commun..

[19] Achan V., Broadhead M., Malaki M., Whitley G., Leiper J., MacAllister R., Vallance P. (2003). Asymmetric Dimethylarginine Causes Hypertension and Cardiac Dysfunction in Humans and Is Actively Metabolized by Dimethylarginine Dimethylaminohydrolase. ATVB.

[20] Murphy R.B., Tommasi S., Lewis B.C., Mangoni A.A. (2016). Inhibitors of the Hydrolytic Enzyme Dimethylarginine Dimethylaminohydrolase (DDAH): Discovery, Synthesis and Development. Molecules.

[21] Jansen K., Hanusch B., Pross S., Hanff E., Drabert K., Bollenbach A., Dugave I., Carmann C., Siefen R.G., Emons B. (2020). Enhanced Nitric Oxide (NO) and Decreased ADMA Synthesis in Pediatric ADHD and Selective Potentiation of NO Synthesis by Methylphenidate. J. Clin. Med..

[22] Kanzelmeyer N., Tsikas D., Chobanyan-Jürgens K., Beckmann B., Vaske B., Illsinger S., Das A.M., Lücke T. (2012). Asymmetric dimethylarginine in children with homocystinuria or phenylketonuria. Amino Acids.

[23] Carmann C., Lilienthal E., Weigt-Usinger K., Schmidt-Choudhury A., Hörster I., Kayacelebi A.A., Beckmann B., Chobanyan-Jürgens K., Tsikas D., Lücke T. (2015). The L-arginine/NO pathway, homoarginine, and nitrite-dependent renal carbonic anhydrase activity in young people with type 1 diabetes mellitus. Amino Acids.

[24] Lücke T., Tsikas D., Kanzelmeyer N., Vaske B., Das A.M. (2006). Elevated plasma concentrations of the endogenous nitric oxide synthase inhibitor asymmetric dimethylarginine in citrullinemia. Metab. Clin. Exp..

[25] Lücke T., Kanzelmeyer N., Kemper M.J., Tsikas D., Das A.M. (2007). Developmental changes in the L-arginine/nitric oxide pathway from infancy to adulthood: Plasma asymmetric dimethylarginine levels decrease with age. Clin. Chem. Lab. Med..

[26] Grasemann H., Al-Saleh S., Scott J.A., Shehnaz D., Mehl A., Amin R., Rafii M., Pencharz P., Belik J., Ratjen F. (2011). Asymmetric dimethylarginine contributes to airway nitric oxide deficiency in patients with cystic fibrosis. Am. J. Respir. Crit. Care Med..

[27] Lucca F., Da Dalt L., Ros M., Gucciardi A., Pirillo P., Naturale M., Perilongo G., Giordano G., Baraldi E. (2018). Asymmetric dimethylarginine and related metabolites in exhaled breath condensate of children with cystic fibrosis. Clin. Respir. J..

[28] Grasemann H., Schwiertz R., Grasemann C., Vester U., Racké K., Ratjen F. (2006). Decreased systemic bioavailability of L-arginine in patients with cystic fibrosis. Respir. Res..

[29] Engelen M.P.K.J., Com G., Luiking Y.C., Deutz N.E.P. (2013). Stimulated nitric oxide production and arginine deficiency in children with cystic fibrosis with nutritional failure. J. Pediatr..

[30] Tsikas D., Böger R.H., Sandmann J., Bode-Böger S.M., Frölich J.C. (2000). Endogenous nitric oxide synthase inhibitors are responsible for the L -arginine paradox. FEBS Lett..

[31] Mels C.M.C., Huisman H.W., Smith W., Schutte R., Schwedhelm E., Atzler D., Böger R.H., Ware L.J., Schutte A.E. (2016). The relationship of nitric oxide synthesis capacity, oxidative stress, and albumin-to-creatinine ratio in black and white men: The SABPA study. AGE.

[32] Grasemann H., Schwiertz R., Matthiesen S., Racké K., Ratjen F. (2005). Increased arginase activity in cystic fibrosis airways. Am. J. Respir. Crit. Care Med..

[33] Oświęcimska J., Ziora K., Ziora D., Machura E., Smerdziński S., Pyś-Spychała M., Kasperski J., Zamłyński J., Kasperska-Zajac A. (2014). Elevated levels of exhaled nitric oxide in patients with anorexia nervosa. Eur. Child Adolesc. Psychiatry.

[34] Rodrigues Pereira N., Bandeira Moss M., Assumpção C.R., Cardoso C.B., Mann G.E., Brunini T.M.C., Mendes-Ribeiro A.C. (2010). Oxidative stress, l-arginine-nitric oxide and arginase pathways in platelets from adolescents with anorexia nervosa. Blood Cells Mol. Dis..

[35] Kromeyer-Hauschild K., Wabitsch M., Kunze D., Geller F., Geiß H.C., Hesse V., von Hippel A., Jaeger U., Johnsen D., Korte W. (2001). Perzentile für den Body-mass-Index für das Kindes- und Jugendalter unter Heranziehung verschiedener deutscher Stichproben. Monatsschr Kinderheilkd.

[36] Neuhauser H., Schienkiewitz A., Rosario A.S., Dortschy R., Kurth B.-M. (2013). Referenzperzentile für anthropometrische Maßzahlen und Blutdruck aus der Studie zur Gesundheit von Kindern und Jugendlichen in Deutschland (KiGGS). Gesundheitsberichterstattung des Bundes.

[37] Malmberg L.P., Petäys T., Haahtela T., Laatikainen T., Jousilahti P., Vartiainen E., Mäkelä M.J. (2006). Exhaled nitric oxide in healthy nonatopic school-age children: Determinants and height-adjusted reference values. Pediatr. Pulmonol..

[38] Tsikas D. (2015). Circulating and excretory nitrite and nitrate: Their value as measures of nitric oxide synthesis, bioavailability and activity is inherently limited. Nitric Oxide.

[39] Tsikas D. (2007). Analysis of nitrite and nitrate in biological fluids by assays based on the Griess reaction: Appraisal of the Griess reaction in the L-arginine/nitric oxide area of research. J. Chromatogr. B Analyt. Technol. Biomed. Life Sci..

[40] Tsikas D. (2011). GC-MS and HPLC methods for peroxynitrite (ONOO- and O15NOO-) analysis: A study on stability, decomposition to nitrite and nitrate, laboratory synthesis, and formation of peroxynitrite from S-nitrosoglutathione (GSNO) and KO2. Analyst.

[41] Tsikas D., Böger R.H., Bode-Böger S.M., Gutzki F.M., Frölich J.C. (1994). Quantification of nitrite and nitrate in human urine and plasma as pentafluorobenzyl derivatives by gas chromatography-mass spectrometry using their 15N-labelled analogs. J. Chromatogr. B Biomed. Appl..

[42] Hanff E., Lützow M., Kayacelebi A.A., Finkel A., Maassen M., Yanchev G.R., Haghikia A., Bavendiek U., Buck A., Lücke T. (2017). Simultaneous GC-ECNICI-MS measurement of nitrite, nitrate and creatinine in human urine and plasma in clinical settings. J. Chromatogr. B Analyt. Technol. Biomed. Life Sci..

[43] Tsikas D., Schubert B., Gutzki F.-M., Sandmann J., Frölich J.C. (2003). Quantitative determination of circulating and urinary asymmetric dimethylarginine (ADMA) in humans by gas chromatography-tandem mass spectrometry as methyl ester tri(N-pentafluoropropionyl) derivative. J. Chromatogr. B Analyt. Technol. Biomed. Life Sci..

[44] Tsikas D., Thum T., Becker T., Pham V.V., Chobanyan K., Mitschke A., Beckmann B., Gutzki F.-M., Bauersachs J., Stichtenoth D.O. (2007). Accurate quantification of dimethylamine (DMA) in human urine by gas chromatography-mass spectrometry as pentafluorobenzamide derivative: Evaluation of the relationship between DMA and its precursor asymmetric dimethylarginine (ADMA) in health and disease. J. Chromatogr. B Analyt. Technol. Biomed. Life Sci..

[45] Bollenbach A., Bakker S.J.L., Tsikas D. (2019). GC-MS measurement of biological NG-hydroxy-L-arginine, a stepmotherly investigated endogenous nitric oxide synthase substrate and arginase inhibitor. Amino Acids.

[46] Bollenbach A., Hanff E., Tsikas D. (2018). Investigation of NG-hydroxy-l-arginine interference in the quantitative determination of nitrite and nitrate in human plasma and urine by GC-NICI-MS. J. Chromatogr. B Analyt. Technol. Biomed. Life Sci..

[47] Tsikas D., Hanff E., Bollenbach A., Kruger R., Pham V.V., Chobanyan-Jürgens K., Wedekind D., Arndt T., Jörns A., Berbée J.F.P. (2018). Results, meta-analysis and a first evaluation of UNOxR, the urinary nitrate-to-nitrite molar ratio, as a measure of nitrite reabsorption in experimental and clinical settings. Amino Acids.

[48] Shwachman H., Kulczycki L.L. (1958). Long-term study of one hundred five patients with cystic fibrosis; studies made over a five- to fourteen-year period. AMA J. Dis. Child..

[49] Chrispin A.R., Norman A.P. (1974). The systematic evaluation of the chest radiograph in cystic fibrosis. Pediatr. Radiol..

[50] Kim S.-O., Corey M., Stephenson A.L., Strug L.J. (2018). Reference percentiles of FEV1 for the Canadian cystic fibrosis population: Comparisons across time and countries. Thorax.

[51] Schlesinger S., Sonntag S.R., Lieb W., Maas R., Shimosawa T. (2016). Asymmetric and Symmetric Dimethylarginine as Risk Markers for Total Mortality and Cardiovascular Outcomes: A Systematic Review and Meta-Analysis of Prospective Studies. PLoS ONE.

[52] Anthony S., Leiper J., Vallance P. (2005). Endogenous production of nitric oxide synthase inhibitors. Vasc. Med..

[53] Vallance P., Leone A., Calver A., Collier J., Moncada S. (1992). Endogenous dimethylarginine as an inhibitor of nitric oxide synthesis. J. Cardiovasc. Pharmacol..

[54] Leiper J.M., Vallance P. (2006). The synthesis and metabolism of asymmetric dimethylarginine (ADMA). Eur. J. Clin. Pharmacol..

[55] Jarzebska N., Mangoni A.A., Martens-Lobenhoffer J., Bode-Böger S.M., Rodionov R.N. (2019). The Second Life of Methylarginines as Cardiovascular Targets. Int. J. Mol. Sci..

[56] Martens-Lobenhoffer J., Bode-Böger S.M. (2014). Mass spectrometric quantification of L-arginine and its pathway related substances in biofluids: The road to maturity. J. Chromatogr. B.

[57] Kerley C.P., Kilbride E., Greally P., Elnazir B. (2016). Dietary Nitrate Acutely and Markedly Increased Exhaled Nitric Oxide in a Cystic Fibrosis Case. Clin. Med. Res..

[58] Closs E.I., Simon A., Vékony N., Rotmann A. (2004). Plasma Membrane Transporters for Arginine. J. Nutr..

[59] Bulau P., Zakrzewicz D., Kitowska K., Leiper J., Gunther A., Grimminger F., Eickelberg O. (2007). Analysis of methylarginine metabolism in the cardiovascular system identifies the lung as a major source of ADMA. Am. J. Physiol. Lung Cell. Mol. Physiol..

[60] Richterich R., Friolet B., Dauwalder H. (1963). Effect of Acetazolamide on Sweat Electrolytes in Mucoviscidosis. JAMA.

[61] Emrich H.M., Ullrich K.J. (1966). Ausscheidung verschiedener Stoffe im Schweiss in Abhängigkeit von der Schweissflussrate. Pflugers Arch. Gesamte Physiol. Menschen Tiere.

[62] Slegers J.F.G., Moons W.M. (1968). Effect of Acetazolamide on the Chloride Shift and the Sodium Pump in Secretory Cells. Nature.

[63] Fanjul M., Salvador C., Alvarez L., Cantet S., Hollande E. (2002). Targeting of carbonic anhydrase IV to plasma membranes is altered in cultured human pancreatic duct cells expressing a mutated (ΔF508) CFTR. Eur. J. Cell Biol..

[64] Ogata T. (2006). Bicarbonate secretion by rat bile duct brush cells indicated by immunohistochemical localization of CFTR, anion exchanger AE2, Na+/HCO3− cotransporter, carbonic anhydrase II, Na+/H+ exchangers NHE1 and NHE3, H+/K+-ATPase, and Na+/K+-ATPase. Med. Mol. Morphol..

[65] Xie C., Tang X., Xu W., Diao R., Cai Z., Chan H.C., Zhou W.-L. (2010). A Host Defense Mechanism Involving CFTR-Mediated Bicarbonate Secretion in Bacterial Prostatitis. PLoS ONE.

[66] Pederzoli A., Mandrioli M., Mola L. (2014). Expression of carbonic anhydrase, cystic fibrosis transmembrane regulator (CFTR) and V-H+-ATPase in the lancelet Branchiostoma lanceolatum (Pallas, 1774). Acta Histochem..

[67] Jantarajit W., Lertsuwan K., Teerapornpuntakit J., Krishnamra N., Charoenphandhu N. (2017). CFTR-mediated anion secretion across intestinal epithelium-like Caco-2 monolayer under PTH stimulation is dependent on intermediate conductance K + channels. Am. J. Physiol. Cell Physiol..

[68] Duranton C., Rubera I., Cougnon M., Melis N., Chargui A., Mograbi B., Tauc M. (2012). CFTR Is Involved in the Fine Tuning of Intracellular Redox Status. Am. J. Pathol..

[69] Krantz C., Janson C., Hollsing A., Alving K., Malinovschi A. (2017). Exhaled and nasal nitric oxide in relation to lung function, blood cell counts and disease characteristics in cystic fibrosis. J. Breath Res..

[70] Umeda M., Hiramoto M., Watanabe A., Tsunoda N., Imai T. (2015). Arginine-induced insulin secretion in endoplasmic reticulum. Biochem. Biophys. Res. Commun..

[71] Kurohane Kaneko Y., Ishikawa T. (2013). Dual role of nitric oxide in pancreatic β-cells. J. Pharmacol. Sci..

[72] Hart N.J., Aramandla R., Poffenberger G., Fayolle C., Thames A.H., Bautista A., Spigelman A.F., Babon J.A.B., DeNicola M.E., Dadi P.K. (2018). Cystic fibrosis–related diabetes is caused by islet loss and inflammation. JCI Insight.

[73] Darville M.I., Eizirik D.L. (1998). Regulation by cytokines of the inducible nitric oxide synthase promoter in insulin-producing cells. Diabetologia.

[74] Novelli M., Pocai A., Lajoix A.D., Beffy P., Bezzi D., Marchetti P., Gross R., Masiello P. (2004). Alteration of β-cell constitutive NO synthase activity is involved in the abnormal insulin response to arginine in a new rat model of type 2 diabetes. Mol. Cell. Endocrinol..

[75] Kelsey R., Manderson Koivula F.N., McClenaghan N.H., Kelly C. (2019). Cystic Fibrosis–Related Diabetes: Pathophysiology and Therapeutic Challenges. Clin. Med. Insights Endocrinol. Diabetes.

[76] Horowitz J.D., Heresztyn T. (2007). An overview of plasma concentrations of asymmetric dimethylarginine (ADMA) in health and disease and in clinical studies: Methodological considerations. J. Chromatogr. B Analyt. Technol. Biomed. Life Sci..

[77] Schnabel R., Blankenberg S., Lubos E., Lackner K.J., Rupprecht H.J., Espinola-Klein C., Jachmann N., Post F., Peetz D., Bickel C. (2005). Asymmetric dimethylarginine and the risk of cardiovascular events and death in patients with coronary artery disease: Results from the AtheroGene Study. Circ. Res..

[78] Hurley M.N., McKeever T.M., Prayle A.P., Fogarty A.W., Smyth A.R. (2014). Rate of improvement of CF life expectancy exceeds that of general population—Observational death registration study. J. Cyst. Fibros..

[79] Skolnik K., Levy R.D., Wilcox P.G., Quon B.S. (2016). Coronary artery disease in cystic fibrosis: An emerging concern?. J. Cyst. Fibros..

[80] Vizzardi E., Sciatti E., Bonadei I., Cani D.S., Menotti E., Prati F., Dallapellegrina L., Metra M., Berlendis M., Poli P. (2019). Macro- and microvascular functions in cystic fibrosis adults without cardiovascular risk factors: A case-control study. Monaldi Arch. Chest Dis..

[81] Labombarda F., Saloux E., Brouard J., Bergot E., Milliez P. (2016). Heart involvement in cystic fibrosis: A specific cystic fibrosis-related myocardial changes?. Respir. Med..

[82] Tsikas D., Bollenbach A., Hanff E., Kayacelebi A.A. (2018). Asymmetric dimethylarginine (ADMA), symmetric dimethylarginine (SDMA) and homoarginine (hArg): The ADMA, SDMA and hArg paradoxes. Cardiovasc. Diabetol..

[83] Beltran-Alvarez P., Pagans S., Brugada R. (2011). The Cardiac Sodium Channel Is Post-Translationally Modified by Arginine Methylation. J. Proteome Res..

[84] Beltran-Alvarez P., Feixas F., Osuna S., Díaz-Hernández R., Brugada R., Pagans S. (2015). Interplay between R513 methylation and S516 phosphorylation of the cardiac voltage-gated sodium channel. Amino Acids.

[85] Beltran-Alvarez P., Tarradas A., Chiva C., Pérez-Serra A., Batlle M., Pérez-Villa F., Schulte U., Sabidó E., Brugada R., Pagans S. (2014). Identification of N-terminal protein acetylation and arginine methylation of the voltage-gated sodium channel in end-stage heart failure human heart. J. Mol. Cell. Cardiol..

[86] Rougé C., Des Robert C., Robins A., Le Bacquer O., Volteau C., La Cochetière M.-F., de Darmaun D. (2007). Manipulation of citrulline availability in humans. Am. J. Physiol. Gastrointest. Liver Physiol..

[87] Chandana T., Venkatesh Y.P. (2016). Occurrence, Functions and Biological Significance of Arginine-Rich Proteins. Curr. Protein Pept. Sci..

[88] Souci S.W., Fachmann W., Kraut H., Andersen G. (2016). Food Composition and Nutrition Tables. Die Zusammensetzung der Lebensmittel, Nährwert-Tabellen.

[89] Kayacelebi A.A., Langen J., Weigt-Usinger K., Chobanyan-Jürgens K., Mariotti F., Schneider J.Y., Rothmann S., Frölich J.C., Atzler D., Choe C.-U. (2015). Biosynthesis of homoarginine (hArg) and asymmetric dimethylarginine (ADMA) from acutely and chronically administered free L-arginine in humans. Amino Acids.

[90] Cloutier M., Gingras D., Bendayan M. (2006). Internalization and transcytosis of pancreatic enzymes by the intestinal mucosa. J. Histochem. Cytochem..

[91] Lozinska L., Prykhodko O., Sureda E.A., Szwiec K., Podgurniak P., Pierzynowski S., Weström B. (2015). Monitoring changes in plasma levels of pancreatic and intestinal enzymes in a model of pancreatic exocrine insufficiency--induced by pancreatic duct-ligation--in young pigs. Adv. Med. Sci..

[92] Isley W.L., Underwood L.E., Clemmons D.R. (1983). Dietary components that regulate serum somatomedin-C concentrations in humans. J. Clin. Investig..

[93] Fontana L., Weiss E.P., Villareal D.T., Klein S., Holloszy J.O. (2008). Long-term effects of calorie or protein restriction on serum IGF-1 and IGFBP-3 concentration in humans. Aging Cell.

[94] Soliman A.T., Hassan A.E., Aref M.K., Hintz R.L., Rosenfeld R.G., Rogol A.D. (1986). Serum insulin-like growth factors I and II concentrations and growth hormone and insulin responses to arginine infusion in children with protein-energy malnutrition before and after nutritional rehabilitation. Pediatr. Res..

[95] Caregaro L., Favaro A., Santonastaso P., Alberino F., Di Pascoli L., Nardi M., Favaro S., Gatta A. (2001). Insulin-like growth factor 1 (IGF-1), a nutritional marker in patients with eating disorders. Clin. Nutr..

[96] Zeng G., Quon M.J. (1996). Insulin-stimulated production of nitric oxide is inhibited by wortmannin. Direct measurement in vascular endothelial cells. J. Clin. Investig..

[97] Darrah R., Bederman I., Vitko M., Valerio D.M., Drumm M.L., Hodges C.A. (2017). Growth deficits in cystic fibrosis mice begin in utero prior to IGF-1 reduction. PLoS ONE.

[98] Pascucci C., de Biase R.V., Savi D., Quattrucci S., Isidori A.M., Lubrano C., Gnessi L., Lenzi A. (2018). Deregulation of the growth hormone/insulin-like growth factor-1 axis in adults with cystic fibrosis. J. Endocrinol. Investig..

[99] Kim M., Lee Y.J., Song H.J., Shim J.K., Chang D.H., Yu W.K., Lee S.-H., Lee J.H. (2017). Supplementation with nutrients modulating insulin-like growth factor-1 negatively correlated with changes in the levels of pro-inflammatory cytokines in community-dwelling elderly people at risk of undernutrition. J. Hum. Nutr. Diet..

[100] Huseman C.A., Colombo J.L., Brooks M.A., Smay J.R., Greger N.G., Sammut P.H., Bier D.M. (1996). Anabolic effect of biosynthetic growth hormone in cystic fibrosis patients. Pediatr. Pulmonol..

[101] Hardin D.S., Sy J.P. (1997). Effects of growth hormone treatment in children with cystic fibrosis: The National Cooperative Growth Study experience. J. Pediatr..

[102] Langen J., Kayacelebi A.A., Beckmann B., Weigt-Usinger K., Carmann C., Hörster I., Lilienthal E., Richter-Unruh A., Tsikas D., Lücke T. (2015). Homoarginine (hArg) and asymmetric dimethylarginine (ADMA) in short stature children without and with growth hormone deficiency: HArg and ADMA are involved differently in growth in the childhood. Amino Acids.

[103] Docx M.K.F., Gewillig M., Simons A., Vandenberghe P., Weyler J., Ramet J., Mertens L. (2010). Pericardial effusions in adolescent girls with anorexia nervosa: Clinical course and risk factors. Eat. Disord..

[104] Inagaki T., Yamamoto M., Tsubouchi K., Miyaoka T., Uegaki J., Maeda T., Horiguchi J., Yamane Y., Kato Y. (2003). Echocardiographic investigation of pericardial effusion in a case of anorexia nervosa. Int. J. Eat. Disord..

[105] Di Cola G., Jacoangeli F., Jacoangeli F., Lombardo M., Iellamo F. (2014). Cardiovascular disorders in anorexia nervosa and potential therapeutic targets. Intern. Emerg. Med..

[106] Miller K.K., Grinspoon S.K., Ciampa J., Hier J., Herzog D., Klibanski A. (2005). Medical findings in outpatients with anorexia nervosa. Arch. Intern. Med..

[107] Shamim T., Golden N., Arden M., Filiberto L., Shenker I. (2003). Resolution of vital sign instability: An objective measure of medical stability in anorexia nervosa. J. Adolesc. Health.

[108] Vignini A., D’Angelo M., Nanetti L., Camilloni M.A., Cester A.M., Faloia E., Salvolini E., Mazzanti L. (2010). Anorexia nervosa: A role for L-arginine supplementation in cardiovascular risk factors?. Int. J. Eat. Disord..

[109] Sachs K.V., Harnke B., Mehler P.S., Krantz M.J. (2016). Cardiovascular complications of anorexia nervosa: A systematic review. Int. J. Eat. Disord..

[110] Mitchell S.C., Zhang A.Q., Smith R.L. (2008). Dimethylamine and diet. Food Chem. Toxicol..

