# Supplementary material for: Activated L-Arginine/Nitric Oxide Pathway in Pediatric Cystic Fibrosis and Its Association with Pancreatic Insufficiency, Liver Involvement and Nourishment: An Overview and New Results
Source: J Clin Med. 2020 Jun 26;9(6):2012. doi: 10.3390/jcm9062012 (PMC7356307; doi:10.3390/jcm9062012)
Supplement: Supplementary file 1 [file jcm-09-02012-s001.pdf]

Journal of
*Clinical Medicine*

MDPI

*Supplement*

# Activated L-arginine/nitric oxide pathway in pediatric cystic fibrosis and its association with pancreatic insufficiency, liver involvement and nourishment: overview and new results

**Folke Brinkmann [1,†], Beatrice Hanusch [1,†,*], Manfred Ballmann [1,2], Sebene Mayorandan [3,4], Alexander Bollenbach [5], Kristine Chobanyan-Jürgens [3,6], Kathrin Jansen [1], Anjona Schmidt-Choudhury [1], Nico Derichs [3,7], Dimitrios Tsikas [5,‡] and Thomas Lücke [1,‡]**

[1] University Children's Hospital, Ruhr University, 44791 Bochum, Germany; folke.brinkmann@rub.de (F.B.); manfred.ballmann@med.uni-rostock.de (M.B.); kathrin.jansen@rub.de (K.J.); a.schmidt-choudhury@klinikum-bochum.de (A.S.-C.); luecke.thomas@rub.de (T.L.)
[2] Current address: Paediatric Clinic, University Medicine Rostock, 18057 Rostock, Germany.
[3] Department of Paediatrics, Hannover Medical School, 30623 Hannover, Germany; Sebene.Mayorandan@ukmuenster.de (S.M.); Kristine.Chobanyan-Juergens@med.uni-heidelberg.de (K.C.-J.); arzt@kinderpneumologie-derichs.de (N.D.)
[4] Current address: Department of Paediatrics, University Clinic Münster, 48149 Münster, Germany.
[5] Institute of Toxicology, Core Unit Proteomics, Hannover Medical School, 30623 Hannover, Germany; bollenbach.alexander@mh-hannover.de (A.B.); tsikas.dimitros@mh-hannover.de (D.T.)
[6] Current address: Department of Clinical Pharmacology and Pharmacoepidemiology, Heidelberg University Hospital, 69120 Heidelberg, Germany, and Department of General Pediatrics, Neuropediatrics, Metabolism, Gastroenterology, Nephrology, Center for Pediatric and Adolescent Medicine, University Hospital Heidelberg, 69120 Heidelberg, Germany, and Pediatric Clinical-Pharmacological Trial Center (paedKliPS), Center for Pediatric and Adolescent Medicine, University Hospital Heidelberg, 69120 Heidelberg, Germany.
[7] Current address: KinderPneumologieDerichs, Pediatric Pneumology and Allergology, CFTR & Pulmonary Research Center, 30173 Hannover, Germany.
* Correspondence: beatrice.hanusch@rub.de; Tel.: +49-234-5092615
† Shared first authors
‡ Shared senior authors

Received: date; Accepted: date; Published: date

**Table S1.** Anthropometric and clinical characteristics of cystic fibrosis (CF) patients

| | **CF patients** |
|---|---|
| Number of subjects (*n*) | 70 |
| Height [percentile] | 36.5 [13.9 – 60.9] |
| BMI [percentile] | 41.8 ± 27.8 |
| Shwachmann-Score | 75.0 [70.0 – 75.0] |
| FEV1 [%] | 90.5 ± 19.1 |
| MEF25 [%] | 59.5 [32.7 – 76.3] |
| Crispin-Norman-Score | 4.5 [3.0 – 9.0] |
| FENO [ppb] | 8.3 [5.9 – 12.9] |
| Fischer-Quotient | 3.3 ± 0.6 |
| Prothrombin time (%) | 84.0 ± 13.2 |
| Cholesterol [mM] | 3.4 ± 0.7 |
| Triglycerides [mM] | 1.1 [0.9 – 1.6] |
| Urea [mM] | 4.4 ± 1.1 |
| Pancreas sufficient [*n* (%)] | 10 (14.3) |
| *P. aeruginosa* negative [*n* (%)] | 49 (70.0) |
| ABPA [*n* (%)] | 6 (8.6) |
| Acute infect [*n* (%)] | 24 (34.3) |
| Steroid treatment [*n* (%)] | 16 (22.9) |
| Liver involvement [*n* (%)] | 8 (11.4) |

Abbreviations. BMI, body-mass-index; FEV1%, Forced expiratory volume in 1 s; MEF25%, mid expiratory flow 25%; FENO, fractional exhaled nitric oxide; ABPA, allergic bronchopulmonary aspergillosis.

Data are reported as median [25-75 percentile] (non-normal distribution) or as mean ± standard deviation (normal distribution).

**Table S2.** Anthropometric and clinical characteristics of cystic fibrosis (CF) patients according to their pancreas sufficiency.

| | **CF patients** | | ***p*** |
|---|---|---|---|
| | Insufficient pancreas | Sufficient pancreas | |
| Number of subjects (*n*) | 60 | 10 | - |
| Height [percentile] | 39.2 [15.0 – 65.7] | 30.8 [11.8 – 44.2] | 0.47 |
| BMI [percentile] | 39.6 ± 27.0 | 55.4 ± 30.2 | 0.10 |
| Shwachmann-Score | 75.0 [70.0 – 75.0] | 75.0 [73.8 – 75.0] | 0.25 |
| FEV1 [%] | 89.6 ± 19.9 | 95.8 ± 13.3 | 0.35 |
| MEF25 [%] | 59.0 [38.0 – 69.5] | 76.0 [30.8 – 113] | 0.24 |
| Crispin-Norman-Score | 4.50 [3.00 – 9.00] | 4.50 [1.75 – 8.50] | 0.58 |
| FENO [ppb] | 8.30 [5.90 – 13.0] | 8.95 [5.33 – 14.7] | 0.93 |
| Fischer-Quotient | 3.35 ± 0.59 | 3.08 ± 0.60 | 0.20 |
| **Prothrombin time (%)** | **82.6 ± 13.0** | **92.3 ± 11.8** | **0.03** |
| **Cholesterol [mM]** | **3.30 ± 0.70** | **4.11 ± 0.50** | **0.01** |
| Triglycerides [mM] | 1.05 [0.84 – 1.56] | 1.19 [1.06 – 1.98] | 0.22 |
| Urea [mM] | 4.45 ± 1.06 | 4.20 ± 1.52 | 0.49$^{\#}$ |
| *P. aeruginosa* negative [n (%)] | 40 (66.7) | 9 (90.0) | 0.26$^{+}$ |
| ABPA [*n* (%)] | 5 (8.30) | 1 (10.0) | 1.00$^{+}$ |
| Acute infect [*n* (%)] | 21 (35.0) | 3 (30.0) | 1.00$^{+}$ |
| Steroid treatment [*n* (%)] | 14 (23.3) | 2 (20.0) | 1.00$^{+}$ |

Abbreviations. BMI, body-mass-index; FENO, fractional exhaled nitric oxide; FEV1%, Forced expiratory volume in 1 s; MEF25%, mid expiratory flow 25%; ABPA, allergic bronchopulmonary aspergillosis.

Data are reported as median [25-75 percentile] (non-normal distribution) or as mean ± standard deviation (normal distribution). $^{\#}$ Unequal variance t-test; $^{+}$ Fisher's exact test; Significant results are marked in bold.

**Table S3.** Anthropometric and clinical characteristics of cystic fibrosis (CF) patients according to the involvement of liver function.

| | **CF patients** | | ***p*** |
|---|---|---|---|
| | Liver involvement | No liver involvement | |
| Number of subjects (*n*) | 8 | 62 | - |
| Height [percentile] | 17.5 [6.90 – 63.7] | 38.8 [18.0 – 60.9] | 0.30 |
| BMI [percentile] | 39.1 (± 26.6) | 42.2 (± 28.2) | 0.77 |
| Shwachmann-Score | 72.5 [70.0 – 75.0] | 75.0 [70.0 – 75.0] | 0.38 |
| FEV1 [%] | 83.4 ± 24.7 | 91.4 ± 18.4 | 0.27 |
| MEF25 [%] | 35.8 [18.0 – 64.3] | 61.0 [38.0 – 80.5] | 0.08 |
| Crispin-Norman-Score | 6.50 [3.00 – 16.0] | 4.00 [2.25 – 9.00] | 0.34 |
| FENO [ppb] | 6.15 [4.00 – 11.7] | 8.50 [6.10 – 13.4] | 0.11 |
| Fischer-Quotient | 3.31 ± 0.57 | 3.31 ± 0.60 | 1.00 |
| **CHE [kU/L]** | **8.11 ± 1.95** | **9.67 ± 1.38** | **0.01** |
| Albumin [g/L] | 41.0 [33.5– 43.0] | 41.0 [39.0 – 43.0] | 0.66 |
| **Prothrombin time (%)** | **73.8 ± 20.4** | **85.34 ± 11.55** | **0.02** |
| **Cholesterin [mM]** | **2.78 ± 0.47** | **3.49 ± 0.72** | **0.02** |
| Triglyceride [mM] | 1.14 [0.85 – 2.27] | 1.07 [0.86 – 1.65] | 0.67 |
| Urea [mM] | 3.70 ± 0.67 | 4.50 ± 1.15 | 0.06 |
| *P. aeruginosa* negative [n (%)] | 5 (62.5) | 44 (71.0) | 0.69$^{+}$ |
| ABPA [*n* (%)] | 0 (0.00) | 6 (9.70) | 1.00$^{+}$ |
| Acute infect [*n* (%)] | 4 (50.0) | 20 (32.3) | 0.43$^{+}$ |
| Steroid treatment [*n* (%)] | 1 (12.5) | 15 (24.2) | 0.67$^{+}$ |

Abbreviations. BMI, body-mass-index; FEV1%, Forced expiratory volume in 1 s; MEF25%, mid expiratory flow 25%; FENO, fractional exhaled nitric oxide; CHE, choline esterase; GFR, glomerular filtration rate; ABPA, allergic bronchopulmonary aspergillosis.

Data are reported as median [25-75 percentile] (non-normal distribution) or as mean ± standard deviation (normal distribution). $^{+}$ Fisher's exact test; Significant results are marked in bold.

**Table S4**. Anthropometric characteristics and biochemical parameters in plasma (P) and urine (U) of the cystic fibrosis patients according to the nutritional status [a]

| | **CF patients** | | ***p*** |
|---|---|---|---|
| | Nutritional failure[a] | Sufficiently nourished[b] | |
| Number of subjects (*n*) | 11 | 59 | - |
| Age [years] | 13.2 [10.2 – 14.2] | 11.1 [8.04 – 14.0] | 0.18 |
| Male [*n* (%)] | 3 (27.3) | 27 (45.8) | 0.31[+] |
| Arg (P) [µM] | 88.2 ± 16.8 | 90.7 ± 21.2 | 0.72 |
| Citrulline (P) [µM] | 33.0 [25.0 – 37.0] | 37.0 [29.8 – 43.0] | 0.22 |
| ADMA (P) [µM] | 0.68 ± 0.11 | 0.60 ± 0.12 | 0.06 |
| **Arg/ADMA (P)** | **130 ± 19.1** | **151 ± 28.2** | **0.02** |
| Nitrate (P) [µM] | 44.9 [37.3 – 58.4] | 42.5 [37.5 – 51.6] | 0.38 |
| Nitrite (P) [µM] | 2.03 [1.85 – 2.34] | 2.08 [1.86 – 2.28] | 0.57 |
| $P_{NOx}R$ | 22.4 [19.4 – 25.7] | 20.7 [16.8 – 24.3] | 0.19 |
| ADMA (U) [µM/mM creatinine] | 8.93 [7.12 – 10.3] | 7.74 [5.69 – 9.85] | 0.21 |
| DMA (U) [µM/mM creatinine] | 56.5 [52.3 – 65.6] | 58.3 [46.4 – 70.9] | 0.93 |
| DMA/ADMA (U) | 7.31 [6.05 – 7.79] | 7.69 [6.42 – 8.98] | 0.19 |
| Nitrat (U) [µM/mM creatinine] | 175 [121 – 275] | 146 [112 – 214] | 0.34 |
| Nitrit (U) [µM/mM creatinine] | 0.21 [0.15 – 0.31] | 0.20 [0.12 – 0.37] | 0.93 |
| $U_{NOx}R$ | 912 ± 404 | 764 ± 376 | 0.24 |

[a]Nutritional failure is defined as a BMI percentile < 10; [b]sufficient nourishment is defined as a BMI percentile ≥ 10 Abbreviations. Arg, arginine; ADMA, asymmetric dimethylarginine; DMA, dimethylamine; P, plasma; U, urine; PNOxR, plasma nitrate/nitrite ratio; UNOxR, urinary nitrate/nitrite ratio.

Data are reported as median [25-75 percentile] (non-normal distribution) or as mean ± standard deviation (normal distribution). Significant results are marked in bold.

**Table S5**. Anthropometric and clinical characteristics of the cystic fibrosis (CF) patients according to their nutritional status.

| | **CF patients** | | ***p*** |
|---|---|---|---|
| | Nutritional failure[a] | Sufficiently nourished[b] | |
| Number of subjects (*n*) | 11 | 59 | - |
| Height [percentile] | 32.9 [10.5 – 53.6] | 40.3 [17.1 – 67.2] | 0.46 |
| **Shwachmann-Score** | **70.0 [65.0 – 75.0]** | **75.0 [70.0 – 75.0]** | **< 0.001** |
| **FEV1 [%]** | **73.0 ± 22.3** | **93.7 ± 16.8** | **0.00** |
| **MEF25 [%]** | **29.0 [21.0 – 63.0]** | **61.0 [38.2 – 82.0]** | **0.04** |
| **Crispin-Norman-Score** | **10.0 [5.00 – 16.0]** | **4.00 [2.00 – 7.50]** | **<0.001** |
| FENO [ppb] | 8.20 [4.70 – 15.7] | 8.30 [5.90 – 12.8] | 0.91 |
| Fischer-Quotient | 3.50 ± 0.85 | 3.27 ± 0.53 | 0.24 |
| Prothrombin time (%) | 81.8 ± 9.38 | 84.4 ± 13.8 | 0.55 |
| Cholesterol [mM] | 3.39 ± 0.57 | 3.40 ± 0.76 | 0.94 |
| Triglycerides [mM] | 1.05 [0.77 – 1.37] | 1.10 [0.86 – 1.85] | 0.47 |
| Urea [mM] | 3.95 ± 0.79 | 4.50 ± 1.17 | 0.15 |
| Pancreas sufficiency [*n* (%)] | 1 (9.10) | 9 (15.2) | 1.00[+] |
| *P. aeruginosa* negative [*n* (%)] | 5 (45.5) | 43 (74.6) | 0.08[+] |
| **ABPA [*n* (%)]** | **3 (27.3)** | **3 (5.10)** | **0.045[+]** |
| Acute infect [*n* (%)] | 6 (54.5) | 18 (30.5) | 0.17[+] |
| Steroid treatment [*n* (%)] | 6 (54.5) | 11 (18.6) | 0.11[+] |
| Liver involvement [*n* (%)] | 1 (9.1) | 7 (11.9) | 1.00 |

[a] Nutritional failure is defined as a BMI percentile < 10; [b] sufficient nourishment is defined as a BMI percentile ≥ 10
Abbreviations. BMIp, BMI percentile; FEV1%, Forced expiratory volume in 1 s; MEF25%, mid expiratory flow 25%;ABPA, allergic bronchopulmonary aspergillosis.

Data are reported as median [25-75 percentile] (non-normal distribution) or as mean ± standard deviation (normal distribution). [+] Fisher's exact test. Significant results are marked in bold.

**Table S6**. Summary of the results of Spearman correlation analyses between members of the Arg/NO pathway in plasma (P) and urine (U) in the cystic fibrosis patients of the study.

| Correlation pair | Correlation coefficient | *p* value | Data pairs (*n*) |
|---|---|---|---|
| Arg (P) vs. Cit (P) | 0.465 | 0.000 | 68 |
| Arg (P) vs. ADMA (P) | 0.636 | 0.000 | 68 |
| Arg (P) vs. $P_{NOx}R$ (P) | 0.240 | 0.049 | 68 |
| Arg/ADMA (P) vs. Cit (P) | 0.345 | 0.004 | 68 |
| Arg (P) vs. ADMA (U) | 0.302 | 0.014 | 68 |
| Arg (P) vs. DMA/ADMA (U) | -0.469 | 0.000 | 66 |
| ADMA (P) vs. Cit (P) | 0.267 | 0.028 | 68 |
| ADMA (P) vs. Nitrite (P) | 0.438 | 0.000 | 66 |
| ADMA (P) vs. $P_{NOx}R$ (P) | 0.300 | 0.013 | 68 |
| ADMA (U) vs. ADMA (P) | 0.438 | 0.000 | 66 |
| DMA/ADMA (U) vs. ADMA (P) | -0.446 | 0.000 | 66 |
| DMA (U) vs. $P_{NOx}R$ | 0.250 | 0.043 | 66 |
| DMA (U) vs. Arg/ADMA (P) | -0.272 | 0.027 | 66 |
| DMA (U) vs. ADMA (U) | 0.661 | 0.000 | 68 |
| Nitrate (U) vs. Nitrate (P) | 0.585 | 0.000 | 66 |
| Nitrate (U) vs. ADMA (U) | 0.561 | 0.000 | 68 |
| Nitrate (U) vs. DMA (U) | 0.618 | 0.000 | 68 |
| Nitrite (U) vs. ADMA (U) | 0.480 | 0.000 | 68 |
| Nitrite (U) vs. DMA (U) | 0.449 | 0.000 | 68 |
| Nitrate (U) vs. $P_{NOx}R$ | 0.439 | 0.000 | 66 |
| Nitrate (U) vs. Nitrite (U) | 0.658 | 0.000 | 68 |
| $U_{NOx}R$ vs. $P_{NOx}R$ | 0.386 | 0.001 | 66 |
| GFR vs. $P_{NOx}R$ | -0.023 | 0.853 | 68 |
| GRF vs. $U_{NOx}R$ | -0.099 | 0.423 | 67 |
| Shwachman-Score vs. Nitrite (P) | 0.255 | 0.040 | 65 |
| Shwachman-Score vs. Arg/ADMA (P) | 0.279 | 0.024 | 65 |

Abbreviations. Arg, arginine; ADMA, asymmetric dimethylarginine; DMA, dimethylamine; P, plasma; U, urine; $P_{NOx}R$, plasma nitrate/nitrite ratio; $U_{NOx}R$, urinary nitrate/nitrite ratio; GFR, glomerular filtration rate.

© 2020 by the authors. Submitted for possible open access publication under the terms and conditions of the Creative Commons Attribution (CC BY) license (http://creativecommons.org/licenses/by/4.0/).
